# Relationships between psychosocial stressors among pregnant women in San Francisco: A path analysis

**DOI:** 10.1371/journal.pone.0234579

**Published:** 2020-06-12

**Authors:** Stephanie M. Eick, Dana E. Goin, Monika A. Izano, Lara Cushing, Erin DeMicco, Amy M. Padula, Tracey J. Woodruff, Rachel Morello-Frosch

**Affiliations:** 1 Department of Obstetrics, Gynecology and Reproductive Sciences, Program on Reproductive Health and the Environment, University of California, San Francisco, San Francisco, California, United States of America; 2 Department of Health Education, San Francisco State University, San Francisco, California, United States of America; 3 Department of Environmental Science, Policy and Management and School of Public Health, University of California, Berkeley, Berkeley, California, United States of America; Chiba Daigaku, JAPAN

## Abstract

Pregnant women who experience psychosocial stressors, such as stressful life events, poor neighborhood quality, and financial hardship, are at an increased risk for adverse pregnancy outcomes. Yet, few studies have examined associations between multiple stressors from different sources, which may be helpful to better inform causal pathways leading to adverse birth outcomes. Using path analysis, we examined associations between multiple self-reported stressor exposures during and before pregnancy in the Chemicals in Our Bodies-2 study (N = 510), a demographically diverse cohort of pregnant women in San Francisco. We examined associations between eight self-reported exposures to stressors and three responses to stress which were assessed via interview questionnaire at the 2^nd^ trimester. Stressors included: neighborhood quality, stressful life events, caregiving, discrimination, financial strain, job strain, food insecurity, and unplanned pregnancy. Perceived stress, depression, and perceived community status were included as indicators of self-reported stress response. Our model indicated that women who experienced discrimination and food insecurity had a 3.76 (95% confidence interval [CI] = 1.60, 5.85) and 2.67 (95% CI = 1.31, 4.04) increase in depression scale scores compared to women who did not experience discrimination and food insecurity, respectively. We additionally identified job strain and caregiving for an ill family member as strong predictors of increased depressive symptoms (β = 1.63, 95% CI = 0.29, 3.07; β = 1.48, 95% CI = 0.19, 2.70, respectively). Discrimination, food insecurity, and job strain also influenced depression indirectly through the mediating pathway of increasing perceived stress, although indirect effects were less precise. In our study population, women who experienced discrimination, food insecurity, job strain and caregiving for an ill family member had an increased number of depressive symptoms compared to women who did not experience these stressors. Results from our study highlight the complex relationships between stressors and stress responses and may help to identify possible mediating pathways leading to adverse pregnancy outcomes.

## Introduction

Studies have shown that exposure to stressors prior to or during pregnancy may increase the likelihood of adverse birth outcomes, including preterm birth and fetal growth restriction [1–4]. There are a number of stressful experiences during pregnancy that have been shown to adversely affect maternal and fetal health, including financial hardship, the death of a family member, or experiences with racial discrimination, and women are often exposed to a multitude of these stressors during pregnancy [5–8]. Certain indicators of psychosocial stress are prevalent during pregnancy, with 39% of women experiencing a stressful life event in the year prior to pregnancy [9] and estimates of depressive symptoms during pregnancy at approximately 25% [10]. Studies have examined diverse psychosocial stressors individually in relation to birth outcomes [11, 12]. However, this may not produce an accurate picture as psychosocial stressors often co-occur and may be linked with one another. For example, individuals who experience stressful life events, such as a family death or trauma, have an increased risk of perceived stress and depression [6, 13, 14]. Similarly, individuals in disadvantaged neighborhoods may be more likely to experience violent crime [15], which may be considered a stressful life event and has been linked to adverse pregnancy outcomes [16]. Women in disadvantaged neighborhoods are also more likely to experience symptoms of depression [17] and have higher risk of preterm birth [18].

Few studies have examined other sources of psychosocial stress, such as caregiving for an ill family member, financial hardship, and job strain, which may also be associated with elevated levels of other stressors and ultimately adverse pregnancy outcomes [19, 20]. For example, it is possible that caregivers experience financial strain, and subsequent depression [21], as a result of missed work. Similarly, unplanned pregnancy has been associated with elevated levels of perceived stress and depression [22, 23].

Psychosocial stress and responses to stress during pregnancy may contribute to the persistence of disparities in adverse birth outcomes across socioeconomic and racial and ethnic groups. For example, research from a multi-center pregnancy cohort indicated that roughly 50% of women with less than a college degree reported experiencing at least one stressful life event during pregnancy, compared to only 33% of women who had completed college [9]. However, stressful life events were not associated with preterm birth in that cohort [24]. Psychosocial stress levels may be higher among lower socioeconomic status (SES) populations due to experiences of greater financial strain, food insecurity, and job strain as compared to higher SES groups [25]. Perceived stress and depression may also be higher among non-white women as a result of racial discrimination [26].

More research is needed to better understand the relationships between multiple stressors to one another and to responses to stress, such as depression, which have been associated with adverse pregnancy outcomes [1]. Additionally, identifying sources of stress may help identify women at a high-risk for adverse outcomes and elucidate better interventions among vulnerable populations. Therefore, the purpose of this study was to evaluate the relationships between psychosocial stressor exposures that include measures such as neighborhood quality, stressful life events, caregiving for an ill family member, discrimination, job strain, food insecurity, financial strain, and unplanned pregnancy, and responses to psychosocial stress, including perceived stress, depression, and perceived community status, in a diverse cohort of pregnant women in San Francisco, CA.

## Material and methods

### Study population

Our study utilized the Chemicals In Our Bodies-2 (CioB) cohort, which recruited pregnant women in their second trimester from the University of California, San Francisco’s Moffit Long, Mission Bay, and Zuckerberg San Francisco General Hospitals during 2014–2018. The Zuckerberg San Francisco General Hospital primarily serves low-income women Medi-Cal (California’s Medicaid program), whereas the Moffit Long and Mission Bay Hospitals serve an economically diverse group of women, the majority of whom have private health insurance. The total sample size for the CiOB cohort was 510 participants, of which 189 women were recruited from Zuckerberg San Francisco General Hospital and 321 women were recruited from either the Moffit Long or Mission Bay Hospitals. The prenatal and delivery hospital at Moffit Long moved to Mission Bay during the study period, however patient populations remain similar demographically. Women were eligible for enrollment if they were >18 years of age, with singleton pregnancies in the second trimester, and spoke English or Spanish as their primary language. In addition to consenting access to their medical records, mothers participated in an interview questionnaire in which they were asked to report their exposures to multiple stressors during and prior to pregnancy. The interview questionnaire is provided in the S1 File. The Institutional Review Boards at the University of California, San Francisco (10–00861) and Berkeley (2010-05-04) approved CiOB and all women provided written, informed consent prior to participating.

Psychosocial stressors and responses to stress were assessed via questionnaire administered by study personnel during a 2^nd^ trimester prenatal care visit. Questionnaires used to assess psychosocial stress and responses to stress have previously validated in other studies. These questionnaires were designed to assess a variety of stress domains that are not routinely examined during pregnancy, including psychosocial stress, work-related stress, and the physical environment.

Psychosocial stressors and responses to stress were derived from the theory of allostasis [27], which has been previously adapted in an effort to better understand and evaluate the effects of pregnancy-related stress, coping, and physiologic changes that may lead to adverse perinatal health outcomes [28]. The theory of allostasis allows us to understand how individuals regulate body systems when they experience expected or unexpected events that are stressful or may be perceived as stressful. We differentiated between stressors and response to stress in our study. Here, stressors were considered to be things that characterize experiences resulting from environmental demands. Responses to stress include those things that may result in adverse maternal and child health outcomes. A detailed description of these measures is summarized in S2 File Table A.

### Psychosocial stressors

Neighborhood quality, stressful life events, caregiving for an ill family member, discrimination, job strain, food insecurity, financial strain, and unplanned pregnancy were included as indicators of self-reported psychosocial stressors in our analysis.

#### Neighborhood quality

Neighborhood quality was assessed using 15 questions related to collective efficacy, neighborhood safety, neighborhood dissatisfaction, and neighborhood physical disorder [29, 30]. Questions that were positively stated were reverse coded and responses to individual questions were summed to create a continuous measure of neighborhood quality. The range of scores on the neighborhood quality scale was between 15 and 69 and higher scores indicate higher stressor levels.

#### Stressful life events

Participants were asked about the occurrences of certain stressful life events within the last 12 months. Stressful life events included close family member hospitalization, separation or divorce from participant’s partner, participant or participant’s partner lost their job, moved to a new address, a close family member experienced immigration problems, participant argued with their partner more than usual, participant’s partner did not want participant to be pregnant, participant was in a physical fight, participant had bills she could not pay, participant’s partner had legal trouble, someone close to participant was drinking or using drugs, someone close to participant passed away. The number of events occurring were summed to create a continuous measure (range 0–11) where higher scores indicate higher stress levels.

#### Caregiving

Participants were asked how often they were responsible for the care and well-being of a parent or older relative or of a child requiring additional medical or educational attention. Responses were ranked on a 5-point scale ranging from “never” to “very often”. If participants answered “often” or “very often” to either questions, they were classified as having experienced caregiving.

#### Discrimination

Discrimination was assessed by asking participants how frequently they felt discriminated against due to their race, ethnicity, religion or color. Responses ranged from “never” to “very often” and were ranked on a 5-point scale. Participants were considered to have experienced discrimination if they answered “often” or “very often”.

#### Job strain

Job strain was assessed using 5 questions which asked participants about how likely they were at their current job to make a lot of decisions, develop their own special abilities, receive a fair salary, do an excessive amount of work, and feel tired and stressed after work. Responses to all questions were ranked on a 5-point scale ranging from “very unlikely” to “very likely”. If participants answered “unlikely” or “very unlikely” to questions regarding decision-making at work, having the opportunity to develop special abilities, and receiving a fair salary, they were considered to have a high strain job. Participants were also considered to have a high strain job if they answered “likely” or “very likely” to questions regarding feeling tired and stressed after work and excessive work load.

#### Food insecurity

Participants were asked if any of the following occurred within the last 12 months as a result of not having enough food: participant or other adults in the household skipped meals, ate less than they felt they should, or were hungry but did not eat. Participants were additionally asked to rate how true it was that they could not afford to eat balanced meals and that the food they had just did not last. Responses to these questions ranged from “never true” to “often true” and were ranked on a 3-point scale. Participants were classified as being food insecure if they reported skipping meals, eating less than they should, or that they were hungry, but did not eat or if they responded “sometimes true” or “often true” to questions about eating balanced meals and not having enough food.

#### Financial strain

Financial strain was assessed by using combined family income and by asking participants how hard it is to pay for basic essentials. Responses ranged from “not difficult” to “very difficult” and were ranked on a 4-point scale. Participants were considered to be under financial strain if their household income was below the San Francisco county poverty line for 2017 or if they reported that it was “somewhat difficult” or “very difficult” to pay for basic necessities such as food, housing, medical care or utilities.

#### Unplanned pregnancy

Participants were asked how they felt about becoming pregnant. Responses ranged from “I didn’t want to be pregnant then” to “I wanted to be pregnant sooner” and were ranked on a 4-point scale. If participants reported wanting to be pregnant later or not wanting to be pregnant at that time, she was considered to have an unplanned pregnancy.

### Responses to psychosocial stress

Perceived stress, depression, and perceived community status were included as possible responses to psychosocial stress in our analyses.

#### Perceived stress

We used the 4-item Perceived Stress Scale (PSS) [31] to measure perceived stress. The PSS is designed to measure the degree to which a participant perceived her life as uncontrollable, unpredictable, and overloading. The total PSS score is a continuous measure where higher scores correspond to higher perceived stress levels. Scores on the PSS in CiOB ranged from 0 to 13.

#### Depression

We used the 10-item Center for Epidemiologic Studies-Depression (CES-D) to measure depression [32]. The CES-D is a clinical screening tool used to measure how often individuals experience depression symptoms in accordance with the Diagnostic Statistical Manual-IV. Higher scores on the CES-D indicate higher depression levels and the range of scores in CiOB was between 0 and 30.

#### Community status

The MacArther Scale of Social Status was used to measure perceived community status [33]. This scale asks participants to place themselves on a continuous scale between 1 and 10 indicating their perceived community standing. Higher scores on the community status scale indicate feelings of lower perceived community status.

### Statistical analysis

**Fig 1:**
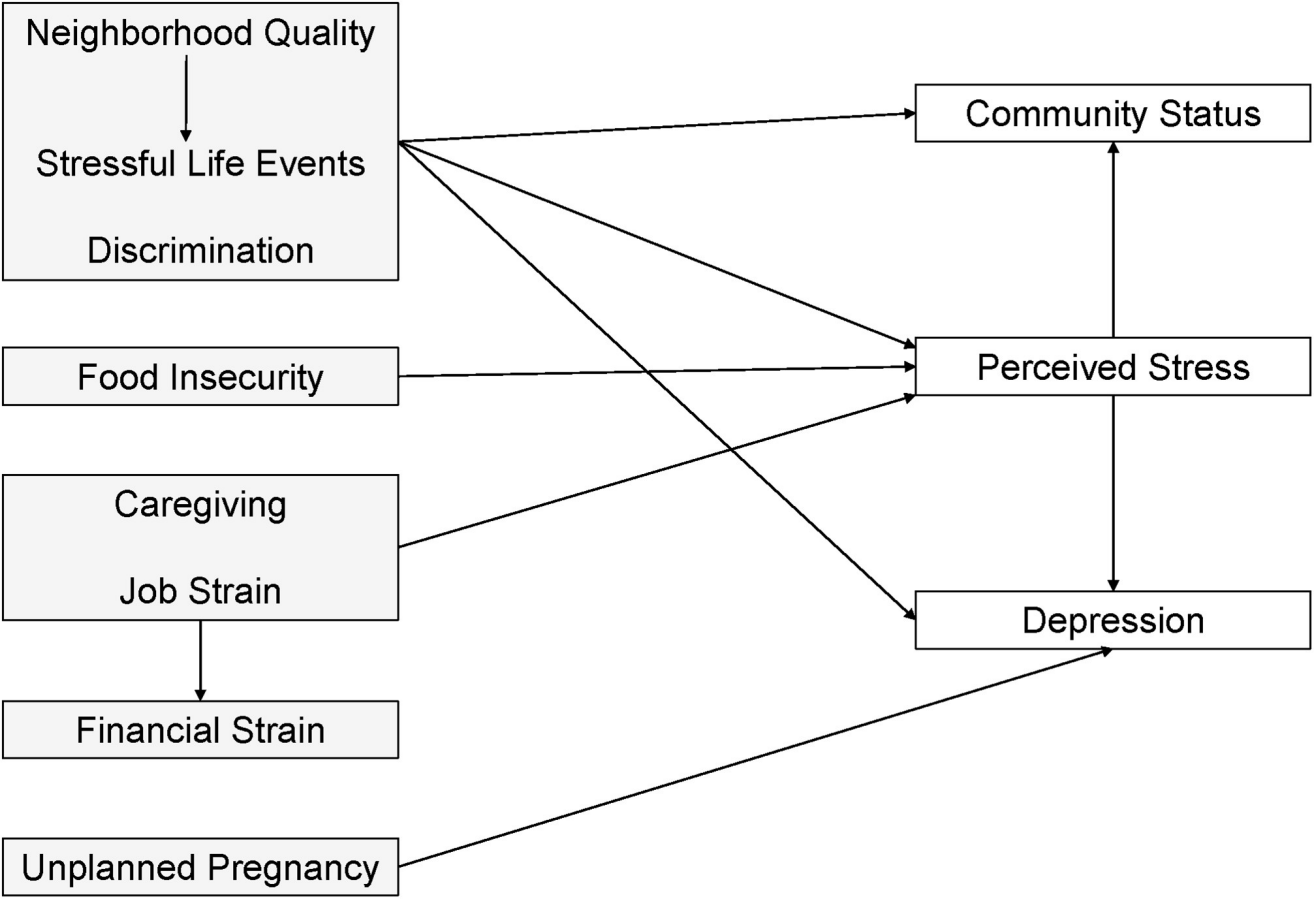
Hypothesized pathways between psychosocial stressors and responses to stress. Gray boxes indicate psychosocial stressor measures and white boxes indicate responses to psychosocial stress.

We examined the distribution of responses to stress and psychosocial stressor measures across demographic characteristics using means, standard deviations (SD), frequencies, and counts. The correlations between psychosocial stress and stress response measures were examined using Spearman’s correlation coefficients for relationships between continuous measures, Pearson’s correlation coefficients for relationships between dichotomous measures, and point biserial correlation coefficients for relationships between continuous and dichotomous measures. Our hypothesized conceptual model of the relationships between psychosocial stress measures was determined through a literature review and is supported by the theory of allostasis (Fig 1).

We conducted a path analysis with full information maximum likelihood (FIML) estimation to test the fit of our hypothesized model using the R package ‘lavaan’ [34]. Path analysis is a subset of structural equation modeling that allows for the estimation of regression coefficients which correspond to the direct, indirect, and total effects among variables. Some key variables were missing data, which motivated our use of FIML, which effectively handles missing data in structural equation models and makes use of all available data by estimating a likelihood function for all participants based on variables that are not missing [35]. Beta estimates in our path analyses are unstandardized regression coefficients and are interpreted analogously to beta estimates obtained from generalized linear models.

The fit of our hypothesized model was tested by first including pre-specified paths. We removed paths that resulted in poor model fit and our hypothesized model was further revised through additional literature review. Model fit was assessed using the Root Mean Square Error of Approximation (RMSEA), Standardized Room Mean Square Residual (SRMR), Comparative Fit Index (CFI), and Tucker Lewis Index (TLI). Values <0.05 and 0.08 indicate good fit for the RMSEA and SRMR, respectively, and values >0.9 indicate good fit for the CFI and TLI [36].

Bootstrapped standard errors and corresponding 95% confidence intervals (CI) were calculated using 1,000 draws. Our final model was *a priori* adjusted for maternal education (≥college degree yes/no), maternal race/ethnicity (non-Hispanic white yes/no), and maternal age at enrollment. These variables were chosen based on their known associations with psychosocial stressors during pregnancy [9].

We conducted a sensitivity analysis to identify SES indicators that might be possible upstream factors leading to elevated psychosocial stressor levels. In the sensitivity analysis, we included education, maternal race/ethnicity, and nativity status (i.e., if the participant was born in the U.S.) as predictors of psychosocial stressors. Said differently, we added education, race/ethnicity, and foreign born as exposure variables to *all* stressor *and* stress response measures. This differed from our first model which included education, race/ethnicity, and maternal age as covariates on pre-existing paths leading to measures of stress response (perceived stress, depression, and community standing) only. In this analysis, we used the weighted least squares means and variance (WLSMV) estimation which allows for dichotomous mediator variables. Complete cases (N = 258) were included in our sensitivity analysis as only complete data is allowed with the WLSMV estimator. All analyses were conducted in R Version 3.6.0 and SAS 9.4.

## Results

The mean age at enrollment was 32 years of age (SD = 5.4 years). The majority of women had either a college degree (23%) or had completed graduate school (36%). A large percentage of women self-identified as non-Hispanic white (38%) or Hispanic (34%) (Table 1). Roughly 15% of our study population reported caregiving for a family member requiring medical or educational attention, 34% experienced financial strain, and 27% of women indicated that their current pregnancy was unplanned (Table 1). The distribution of self-reported psychosocial stressor measures and responses across racial and ethnic groups and demographic characteristics is presented in S2 File Tables B and C, respectively.

**Table 1:** Distribution of demographic characteristics and psychosocial stress measures and responses to stress in the Chemicals in Our Bodies cohort (N = 510).

	N (%) or Mean (SD)
**Maternal Age at Enrollment**	
Mean (SD)	32 (5.4)
Missing	1 (0.2%)
**Maternal Education**	
Less than High School	59 (12%)
High School Degree or Some College	140 (27%)
College Degree	118 (23%)
Graduate Degree	185 (36%)
Missing	8 (1.6%)
**Maternal Race/Ethnicity**	
Non-Hispanic White	194 (38%)
Non-Hispanic Black	41 (8.0%)
Hispanic	174 (34%)
Asian/Pacific Islander	95 (19%)
Missing	6 (1.2%)
**Marital Status**	
Married	337 (66%)
Single	161 (32%)
Missing	12 (2.4%)
**Pre-pregnancy Body Mass Index**	
Underweight (<18.5 kg/m^2^)	12 (2.0%)
Normal Weight (18.5–24.9 kg/m^2^)	237 (46%)
Overweight (25.0–29.9 kg/m^2^)	129 (25%)
Obese (≥30 kg/m^2^)	90 (18%)
Missing	42 (8.2%)
**Parity**	
One or More Prior Births	247 (48%)
Missing	8 (1.6%)
**Foreign Born**	
Yes	210 (41%)
Missing	86 (17%)
**Perceived Stress**^a^ (range 0–13)	
Mean (SD)	5.4 (2.7)
Missing	17 (3.3)
**Depression**^a^ (range 0–30)	
Mean (SD)	7.3 (5.2)
Missing	39 (7.6)
**Neighborhood Quality**^a^ (range 15–69)	
Mean (SD)	40 (9.5)
Missing	84 (16.5)
**Community Status**^a^ (range 1–10)	
Mean (SD)	6.4 (1.9)
Missing	42 (8.2)
**Stressful Life Events** (range 0–11)	
Mean (SD)	2.1 (1.8)
Missing	11 (2.2)
**Caregiving**	
Yes	78 (15%)
Missing	13 (2.5%)
**Discrimination**	
Yes	35 (7%)
Missing	17 (3.3%)
**Financial Strain**	
Yes	174 (34%)
Missing	65 (12.7%)
**Job Strain**	
Yes	65 (13%)
Missing	47 (9.2%)
**Unplanned Pregnancy**	
Yes	139 (27%)
Missing	16 (3.1%)
**Food Insecurity**	
Yes	81 (16%)
Missing	11 (2.2%)

^a^Higher scores for Perceived Stress, Depression, and Neighborhood Quality indicate higher stressor and response levels. Lower scores for Community Status indicate higher stress response levels.

Abbreviations: SD, standard deviation.

Correlation coefficients between psychosocial stressors and response to stress measures are presented in Table 2. The strongest correlations were between discrimination and depression (point biserial *r* = 0.75), food insecurity and depression (point biserial *r* = 0.65), and perceived stress and depression (Spearman’s *r* = 0.60). Perceived community status was negatively correlated with all measures, as expected.

**Table 2:** Correlation coefficients between continuous psychosocial stress measures (stressful life events, neighborhood quality, discrimination, food insecurity, caregiving, job strain, financial strain, unplanned pregnancy) and responses to stress (depression, perceived stress, community status).

	Depression	Perceived Stress	Community Status	Stressful Life Events	Neighborhood Quality	Discrimination	Food Insecurity	Caregiving	Job Strain	Financial Strain	Unplanned Pregnancy
Depression	1										
Perceived Stress	0.60^a^	1									
Community Status	-0.16^a^	-0.17^a^	1								
Stressful Life Events	0.40^a^	0.35^a^	-0.12^a^	1							
Neighborhood Quality	0.20^a^	0.24^a^	-0.22^a^	0.25^a^	1						
Discrimination	0.75^b^	0.46^b^	-0.32^b^	0.59^b^	0.34^b^	1					
Food Insecurity	0.65^b^	0.46^b^	-0.26^b^	0.47^b^	0.33^b^	0.24^c^	1				
Caregiving	0.48^b^	0.26^b^	-0.13^b^	0.41^b^	0.18^b^	0.27^c^	0.24^c^	1			
Job Strain	0.39^b^	0.26^b^	-0.26^b^	0.17^b^	0.35^b^	0.07^c^	0.24^c^	0.15^c^	1		
Financial Strain	0.50^b^	0.50^b^	-0.38^b^	0.46^b^	0.39^b^	0.21^c^	0.54^c^	0.34^c^	0.36^c^	1	
Unplanned Pregnancy	0.33^b^	0.31^b^	-0.21^b^	0.38^b^	0.19^b^	0.09^c^	0.24^c^	0.22^c^	0.22^c^	0.33^c^	1

Higher scores for Perceived Stress, Depression, and Neighborhood Quality indicate higher stressor and response levels. Lower scores for Community Status indicate higher stress response levels.

^a^Indicates Spearman correlation coefficient.

^b^Indicates point biserial correlation coefficient.

^c^Indicates Pearson correlation coefficient.

**Fig 2:**
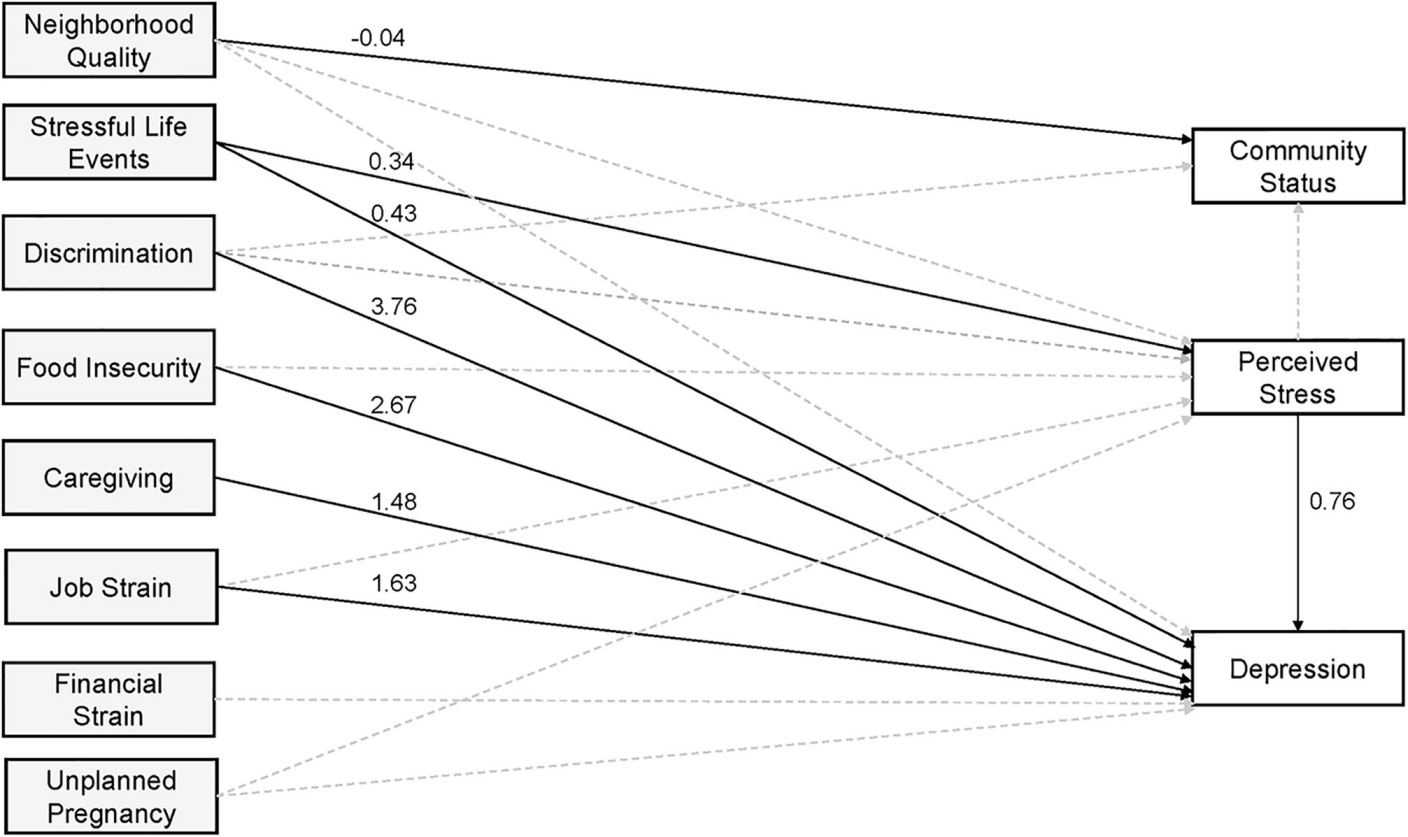
Full empirical model indicating the associations between psychosocial stressors and responses to stress. Overall model had good fit: RMSEA = 0.04, SRMR = 0.01, CFI = 0.99, TLI = 0.96. Model is adjusted for maternal age, maternal education, and maternal race/ethnicity. Solid black lines indicate statistically significant paths at p<0.05. Gray dashed lines indicate non-significant paths. Effect estimates correspond to path coefficients for the direct effect provided in Table 3. Gray boxes indicate psychosocial stress measures and white boxes indicate responses to psychosocial stress. Higher scores for Perceived Stress, Depression, and Neighborhood Quality indicate higher stressor and response levels. Lower scores for Community Status indicate higher stress response levels.

Our model of the relationships between multiple psychosocial stressors and responses to stress is shown in Fig 2 and beta estimates and 95% CIs are presented in Table 3. Model fit was determined to be good (RMSEA = 0.04, SRMR = 0.01, CFI = 0.99, TLI = 0.96). Increased stressful life events (β = 0.43, 95% CI = 0.15, 0.70), perceived stress (β = 0.76, 95% CI = 0.59, 0.96), and experiences of discrimination (β = 3.76, 95% CI = 1.60, 5.85), caregiving (β = 1.48, 95% CI = 0.19, 2.70), food insecurity (β = 2.67, 95% CI = 1.31, 4.04), and job strain (β = 1.63, 95% CI = 0.29, 3.07) were directly associated with higher depression scores, controlling for age, race/ethnicity and education. Perceived stress also mediated the association between stressful life events and depression (β = 0.26, 95% CI = 0.14, 0.40). Poor neighborhood quality, unplanned pregnancy, and financial strain were not directly nor indirectly associated with depression symptoms (Table 3).

**Table 3:** Regression coefficients and 95% confidence intervals for direct, indirect, and total effects between psychosocial stress measures in the Chemicals in Our Bodies-2 cohort (N = 510). Model has good fit (RMSEA = 0.04, SRMR = 0.01, CFI = 0.99, TLI = 0.96).

			Direct Effect		Indirect Effect		Total Effect	
Independent Variable	Dependent Variable	Mediator Variable	Beta	95% CI	Beta	95% CI	Beta	95% CI
Perceived Stress^a^	Depression^a^	–	**0.76**	**(0.59, 0.96)**	–	–	**0.76**	**(0.59, 0.96)**
Neighborhood Quality^a^		Perceived Stress^a^	-0.01	(-0.05, 0.04)	0.02	(0.00, 0.04)	0.01	(-0.04, 0.06)
Stressful Life Events^a^		Perceived Stress^a^	**0.43**	**(0.15, 0.70)**	**0.26**	**(0.14, 0.40)**	**0.69**	**(0.40, 0.99)**
Discrimination^b^		Perceived Stress^a^	**3.76**	**(1.60, 5.85)**	0.71	(-0.13, 1.65)	**4.47**	**(2.31, 6.65)**
Food Insecurity^b^		Perceived Stress^a^	**2.67**	**(1.31, 4.04)**	0.40	(-0.13, 1.06)	**3.07**	**(1.60, 4.56)**
Caregiving^b^		–	**1.48**	**(0.19, 2.70)**	–	–	**1.48**	**(0.19, 2.70)**
Job Strain^b^		Perceived Stress^a^	**1.63**	**(0.29, 3.07)**	0.22	(-0.35, 0.91)	**1.85**	**(0.32, 3.57)**
Financial Strain^b^		–	-0.36	(-1.71, 0.89)	–	–	-0.36	(-1.71, 0.89)
Unplanned Pregnancy^b^		Perceived Stress^a^	0.72	(-0.32, 1.64)	0.33	(-0.03, 0.73)	1.05	(-0.09, 2.05)
Neighborhood Quality^a^	Perceived Stress^a^	–	0.03	(0.00, 0.05)	–	–	0.03	(0.00, 0.05)
Stressful Life Events^a^		–	**0.34**	**(0.20, 0.49)**	–	–	**0.34**	**(0.20, 0.49)**
Discrimination^b^		–	0.93	(-0.17, 2.03)	–	–	0.93	(-0.17, 2.03)
Food Insecurity^b^		–	0.52	(-0.17, 1.33)	–	–	0.52	(-0.17, 1.33)
Job Strain^b^		–	0.29	(-0.46, 1.15)	–	–	0.29	(-0.46, 1.15)
Unplanned Pregnancy^b^		–	0.44	(-0.04, 0.94)	–	–	0.44	(-0.04, 0.94)
Perceived Stress^a^	Community Status^a^	–	-0.04	(-0.12, 0.03)	–	–	-0.04	(-0.12, 0.03)
Neighborhood Quality^a^		Perceived Stress^a^	**-0.04**	**(-0.06, -0.02)**	0.00	(0.00, 0.00)	**-0.04**	**(-0.06, -0.02)**
Stressful Life Events^a^		Perceived Stress^a^	–	–	-0.01	(-0.04, 0.01)	-0.01	(-0.04, 0.01)
Discrimination^b^		Perceived Stress^a^	-0.71	(-1.66, 0.27)	-0.04	(-0.17, 0.03)	-0.75	(-1.67, 0.21)
Food Insecurity^b^		Perceived Stress^a^	–	–	-0.02	(-0.10, 0.02)	-0.02	(-0.10, 0.02)
Job Strain^b^		Perceived Stress^a^	–	–	-0.01	(-0.08, 0.03)	-0.01	(-0.08, 0.03)
Unplanned Pregnancy^b^		Perceived Stress^a^	–	–	-0.02	(-0.07, 0.02)	-0.02	(-0.07, 0.02)

Model adjusted for maternal age, maternal race (non-Hispanic white yes/no), and maternal education (<college degree, college or graduate degree). Higher scores for Perceived Stress, Depression, and Neighborhood Quality indicate higher stressor and response levels. Lower scores for Community Status indicate higher stress response levels. Bold indicates statistical significance at p<0.05.

Abbreviations: CI, confidence interval.

-Indicates no path.

^a^Indicates continuous measure.

^b^Indicates dichotomous measure.

An increased number of stressful life events (β = 0.34, 95% CI = 0.20, 0.49) was positively associated with perceived stress through a direct path. Discrimination and poor neighborhood quality were also moderately associated with higher perceived stress levels. With the exception of poor neighborhood quality, none of the other psychosocial stressors were associated with perceived community status in our final model (Table 3). Contrary to our hypotheses, neighborhood quality was not a predictor of stressful life events and caregiving was not a predictor of perceived stress. Additionally, caregiving and job strain were not associated with financial strain in our model.

Effect estimates for our sensitivity analyses including SES indicators as upstream predictors of stressors is provided in S2 File Tables D–H. Our sensitivity analysis indicated that having less than a college education was directly associated with experiencing stressful life events, discrimination, poor neighborhood quality, food insecurity, job strain, unplanned pregnancy, caregiving, and financial strain (S2 File **Table E;** S3 File **Fig A**). Maternal race/ethnicity was directly associated with financial strain and food insecurity (S2 File **Table E;** S3 File **Fig A**). Being foreign-born was directly associated with lower perceived community status but no other psychosocial stressors (S2 File **Table E;** S3 File **Fig A**). Indirect and total effects of SES indicators on indicators of stress response (perceived stress, depression, community status) mediated by psychosocial stressors (stressful life events, discrimination, neighborhood quality, food insecurity, job strain, unplanned pregnancy, caregiving, financial strain) are presented in S2 File **Tables F–H**. Associations between psychosocial stressors and responses to stress were similar in our sensitivity analysis as compared to our main analysis, although point estimates were somewhat stronger in our sensitivity analysis (S2 File **Table D**).

## Discussion

Our study set out to better understand the associations between multiple self-reported stressors and stress response outcomes, many of which previously have not been examined together. We created a conceptual model to indicate the relationships between multiple psychosocial stressors and responses to stress. Our model was tested using a path analysis, which allowed us to elucidate the pathways between stressors and measures of stress responses and we identified caregiving, discrimination, food insecurity, job strain, and unplanned pregnancy as important predictors of increased depressive symptoms. We additionally observed that individuals experiencing stressful life events had higher levels of depression and identified maternal education as an upstream SES indicator associated with increased stressful life events. Results from this study also suggest that women who experience stressful life events have higher levels of perceived stress.

Our findings linking stressful life events and perceived stress to depressive symptoms are supported by previous studies of pregnant women. A cross-sectional study of women receiving prenatal care in Virginia showed that elevated perceived stress levels were associated with increased odds of depression [13]. These findings were confirmed by a prospective cohort of pregnant women in Iran, which additionally showed that depression symptoms were elevated subsequent to experiencing stressful life events [14]. Additionally, stressful life events were linked to increased odds of postpartum depression, with the strongest effects observed for relationship related stressors, within the Mississippi Pregnancy Risk Assessment Monitoring System [6]. Lastly, a prospective cohort study in Puerto Rico found that an increasing number of stressful life events was associated with increased feelings of perceived stress and symptoms of depression [7]. In that study, the indirect effect of stressful life events on depression through increasing perceived stress was stronger than the direct effect [7].

We identified perceived discrimination as a strong predictor of depressive symptoms during pregnancy. This finding is consistent with previous research conducted among pregnant women in the Czech Republic which showed that perceived discrimination was associated with increased odds of postpartum depression [37]. Similarly, a population-based study of young (ages 18–20) women in the Midwest region of the U.S. found a positive association between discrimination and depressive symptoms [38]. That study also found a positive association between discrimination and perceived stress [38], which was not observed in our study. Differences may be attributed to differences in study populations, as our study was conducted among pregnant women who were slightly older.

We found no association between unplanned pregnancy and perceived stress or depression in our study, which contrasts with our hypothesis and prior studies. For example, a prospective cohort study of women in the Netherlands found that women who reported having an unplanned pregnancy had higher scores on the depression subscale of the Hospital Anxiety and Depression Scale (HADS) during early pregnancy [39]. However, associations between unplanned pregnancy and depression were attenuated to non-significance when the HADS was administered in late pregnancy, which is consistent with our findings.

Our study identified food insecurity as an upstream predictor of depression. However, we observed no associations between food insecurity and perceived stress. Elevated postpartum perceived stress scores were observed among women in North Carolina who reported being food insecure during pregnancy [40]. It is possible that we did not observe an association between food insecurity and perceived stress as a result of when perceived stress was measured, which was during pregnancy, not postpartum. Furthermore, there may be other SES factors, such as poverty and a lack of affordable housing in the Bay Area, which could be influencing food insecurity in our study population and may explain these differences. Nonetheless, our finding that food insecurity was associated with higher scores on the depression scale is supported by a cross-sectional study conducted among pregnant women in South Africa [41].

Poor neighborhood quality was not associated with perceived stress or depressive symptoms in our cohort, which is in contrast to previous work. For example, a study among African American pregnant women in Detroit found that lower neighborhood quality, as measured by indicators of social and physical disorder, safety, walking environment, and overall neighborhood quality, was associated with elevated depression symptoms and that the association was partially mediated by perceived stress [42]. A second study found that lower perceived neighborhood safety and walkability was associated with increased depression symptoms during pregnancy among African American women [43]. Discrepancies in our results may be a result of how neighborhood quality was measured. Neighborhood quality was a composite measure of collective efficacy, neighborhood safety, neighborhood dissatisfaction, and neighborhood physical disorder, which is somewhat different than other studies. Differences may also be due to differences in study populations, as less than 10% of our study population identified as being non-Hispanic black. Lastly, San Francisco experienced a severe shortage of affordable housing crisis and rapid gentrification during our study period, which presents unique challenges and may have resulted in differences in how one’s neighborhood is perceived that may be specific to the San Francisco Bay Area.

Previous studies have also linked financial strain to depression, which was not observed in our study [23, 44]. Among a pregnancy cohort in Boston, postpartum experiences of financial hardship were associated with increased odds of postpartum depressive symptoms [23]. Financial strain and depression were assessed during pregnancy, not postpartum, in our study, which could explain these differences. An additional study among women of child bearing age in the United Kingdom found that women experiencing financial hardship had increased hazards of depression, these associations were attenuated to non-significance after adjusting for covariates, which is consistent with our findings [44].

Our study has a several strengths. First, we included multiple indicators of psychosocial stress, which allowed us to determine which stressors may be strongly linked to responses to stress, including depression, a clinical outcome with implications for maternal and infant health. This is a vital expansion on prior research which often only included a small number of stress measures. The CiOB cohort also includes women from a variety of racial and ethnic groups and many SES levels, an important advancement over previous work that have been conducted among racially homogenous populations and high SES populations [42, 45]. Additionally, information regarding psychosocial stress was collected prior to delivery, which reduces the likelihood that women would have reported higher levels of stress as a result of an adverse pregnancy outcome. Lastly, the theory of allostasis was used to inform how we grouped psychosocial stressors and responses to psychosocial stress in this analysis. Our findings are also supported by the social ecological model, which acknowledges that multiple levels, such as psychosocial states, communities, and the built environment, influence and impact the health and behavior of individuals.

We also acknowledge several limitations of this study. Our cohort was not a random sample, and therefore our results may not be generalizable to populations beyond the study sample. Additionally, this was an exploratory analysis conducted within an existing study that was designed to address additional research questions. Measures of psychosocial stress were obtained at the same study visit and in that sense our study may be considered cross-sectional. It is possible that women may have been more likely to report experiencing financial strain or job strain as a result of pre-existing depression, rather than financial or job strain leading to depression, and we are unable to differentiate between those pathways here. Lastly, we may have been limited by a small sample size as some of our psychosocial stress measures, such as job strain and discrimination, occurred in a relatively small number of participants.

## Conclusions

Among a diverse cohort of pregnant women in San Francisco, we identified caregiving, discrimination, food insecurity, job strain, and unplanned pregnancy as important upstream stressors associated with depression. It is imperative to understand the mechanisms by which stressors influence one another to better identify specific causal pathways leading to adverse maternal and child health outcomes. Findings from this study may help clinicians in identifying women who may be at an increased risk for adverse birth outcomes. Importantly, many of the stressors examined in this analysis have been associated with adverse birth outcomes, including preterm birth, in other studies [1, 2, 11]. Our findings also identify vulnerable populations and can inform interventions aimed at alleviating food insecurity, job strain, and discrimination, and reducing the burden of depression during pregnancy. Future studies that map relationships between multiple self-reported stressor exposures and responses could be strengthened by also integrating biological measures of stress response, which may be elevated in response to some of the self-reported exposures examined here, and could better characterizing the indirect pathways we identified.

## Supporting information

S1 File Interview questionnaire used to assess psychosocial stressors and responses to psychosocial stressors in CiOB. (PDF)

S2 File Supporting tables for relationships between psychosocial stressors among pregnant women in San Francisco: A path analysis. (DOCX)

S3 File Supporting figure for relationships between psychosocial stressors among pregnant women in San Francisco: A path analysis. (TIFF)

## References

[1] Liu C, Cnattingius S, Bergström M, Östberg V, Hjern A. Prenatal parental depression and preterm birth: a national cohort study. Bjog. 2016;123(12):1973–82. Epub 2016/10/19. 10.1111/1471-0528.13891 26786413 PMC5096244

[2] Shapiro GD, Fraser WD, Frasch MG, Séguin JR. Psychosocial stress in pregnancy and preterm birth: associations and mechanisms. J Perinat Med. 2013;41(6):631–45. Epub 2013/11/13. 10.1515/jpm-2012-0295 24216160 PMC5179252

[3] Lilliecreutz C, Larén J, Sydsjö G, Josefsson A. Effect of maternal stress during pregnancy on the risk for preterm birth. BMC Pregnancy and Childbirth. 2016;16(1):5 10.1186/s12884-015-0775-x 26772181 PMC4714539

[4] Sabri Y, Nabel H. The impact of anxiety and depression during pregnancy on fetal growth and the birth outcome. Egyptian Journal of Psychiatry. 2015;36(2):95–100. 10.4103/1110-1105.158117

[5] Lima SAM, El Dib RP, Rodrigues MRK, Ferraz GAR, Molina AC, Neto CAP, et al Is the risk of low birth weight or preterm labor greater when maternal stress is experienced during pregnancy? A systematic review and meta-analysis of cohort studies. PLoS One. 2018;13(7):e0200594 Epub 2018/07/27. 10.1371/journal.pone.0200594 30048456 PMC6061976

[6] Qobadi M, Collier C, Zhang L. The Effect of Stressful Life Events on Postpartum Depression: Findings from the 2009–2011 Mississippi Pregnancy Risk Assessment Monitoring System. Matern Child Health J. 2016;20(Suppl 1):164–72. Epub 2016/06/25. 10.1007/s10995-016-2028-7 27339648 PMC5290058

[7] Eick SM, Meeker JD, Swartzendruber A, Rios-McConnell R, Brown P, Vélez-Vega C, et al Relationships between psychosocial factors during pregnancy and preterm birth in Puerto Rico. PloS one. 2020;15(1):e0227976–e. 10.1371/journal.pone.0227976 . 31995596 PMC6988967

[8] Alhusen JL, Bower KM, Epstein E, Sharps P. Racial Discrimination and Adverse Birth Outcomes: An Integrative Review. J Midwifery Womens Health. 2016;61(6):707–20. Epub 2016/10/14. 10.1111/jmwh.12490 27737504 PMC5206968

[9] Eick SM, Barrett ES, van ’t Erve TJ, Nguyen RHN, Bush NR, Milne G, et al Association between prenatal psychological stress and oxidative stress during pregnancy. Paediatr Perinat Epidemiol. 2018;32(4):318–26. Epub 2018/04/01. 10.1111/ppe.12465 29603338 PMC6103836

[10] Da Costa D, Larouche J, Dritsa M, Brender W. Psychosocial correlates of prepartum and postpartum depressed mood. J Affect Disord. 2000;59(1):31–40. Epub 2000/05/18. 10.1016/s0165-0327(99)00128-7 . 10814768

[11] Dole N, Savitz DA, Hertz-Picciotto I, Siega-Riz AM, McMahon MJ, Buekens P. Maternal Stress and Preterm Birth. American Journal of Epidemiology. 2003;157(1):14–24. 10.1093/aje/kwf176 12505886

[12] Zhang S, Ding Z, Liu H, Chen Z, Wu J, Zhang Y, et al Association between mental stress and gestational hypertension/preeclampsia: a meta-analysis. Obstet Gynecol Surv. 2013;68(12):825–34. Epub 2014/08/08. 10.1097/OGX.0000000000000001 . 25102019

[13] Kinser PA, Thacker LR, Lapato D, Wagner S, Roberson-Nay R, Jobe-Shields L, et al Depressive Symptom Prevalence and Predictors in the First Half of Pregnancy. J Womens Health (Larchmt). 2018;27(3):369–76. Epub 2017/12/15. 10.1089/jwh.2017.6426 29240527 PMC5865242

[14] Dolatian M, Mahmoodi Z, Alavi-Majd H, Moafi F, Ghorbani M, Mirabzadeh A. Psychosocial factors in pregnancy and birthweight: Path analysis. J Obstet Gynaecol Res. 2016;42(7):822–30. Epub 2016/04/22. 10.1111/jog.12991 . 27098096

[15] Krivo LJ, Peterson RD. Extremely Disadvantaged Neighborhoods and Urban Crime*. Social Forces. 1996;75(2):619–48. 10.1093/sf/75.2.619

[16] Goin DE, A MG, Farkas K, Zimmerman SC, Matthay EC, Ahern J. Exposure to Community Homicide During Pregnancy and Adverse Birth Outcomes: A Within-Community Matched Design. Epidemiology. 2019;30(5):713–22. Epub 2019/06/11. 10.1097/EDE.0000000000001044 31180933 PMC6677586

[17] Barcelona de Mendoza V, Harville EW, Savage J, Giarratano G. Experiences of Intimate Partner and Neighborhood Violence and Their Association With Mental Health in Pregnant Women. J Interpers Violence. 2018;33(6):938–59. Epub 2015/11/19. 10.1177/0886260515613346 26576616 PMC4870174

[18] Ncube CN, Enquobahrie DA, Albert SM, Herrick AL, Burke JG. Association of neighborhood context with offspring risk of preterm birth and low birthweight: A systematic review and meta-analysis of population-based studies. Soc Sci Med. 2016;153:156–64. Epub 2016/02/24. 10.1016/j.socscimed.2016.02.014 . 26900890 PMC7302006

[19] Mitchell AM, Christian LM. Financial strain and birth weight: the mediating role of psychological distress. Arch Womens Ment Health. 2017;20(1):201–8. Epub 2016/12/14. 10.1007/s00737-016-0696-3 27957597 PMC5239729

[20] Croteau A, Marcoux S, Brisson C. Work Activity in Pregnancy, Preventive Measures, and the Risk of Preterm Delivery. American Journal of Epidemiology. 2007;166(8):951–65. 10.1093/aje/kwm171 17652310

[21] Kim D. Relationships between Caregiving Stress, Depression, and Self-Esteem in Family Caregivers of Adults with a Disability. Occup Ther Int. 2017;2017:1686143 Epub 2017/11/09. 10.1155/2017/1686143 29114184 PMC5664279

[22] Nelson DB, Lepore SJ. The role of stress, depression, and violence on unintended pregnancy among young urban women. J Womens Health (Larchmt). 2013;22(8):673–80. Epub 2013/06/25. 10.1089/jwh.2012.4133 23789582 PMC3736642

[23] Rich-Edwards JW, Kleinman K, Abrams A, Harlow BL, McLaughlin TJ, Joffe H, et al Sociodemographic predictors of antenatal and postpartum depressive symptoms among women in a medical group practice. J Epidemiol Community Health. 2006;60(3):221–7. Epub 2006/02/16. 10.1136/jech.2005.039370 16476752 PMC2465548

[24] Ferguson KK, Rosen EM, Barrett ES, Nguyen RHN, Bush N, McElrath TF, et al Joint impact of phthalate exposure and stressful life events in pregnancy on preterm birth. Environment International. 2019;133:105254 10.1016/j.envint.2019.105254. 31675562 PMC6924167

[25] Okechukwu CA, El Ayadi AM, Tamers SL, Sabbath EL, Berkman L. Household food insufficiency, financial strain, work-family spillover, and depressive symptoms in the working class: the Work, Family, and Health Network study. Am J Public Health. 2012;102(1):126–33. Epub 2011/11/19. 10.2105/AJPH.2011.300323 22095360 PMC3490565

[26] Bécares L, Atatoa-Carr P. The association between maternal and partner experienced racial discrimination and prenatal perceived stress, prenatal and postnatal depression: findings from the growing up in New Zealand cohort study. International Journal for Equity in Health. 2016;15(1):155 10.1186/s12939-016-0443-4 27658457 PMC5034520

[27] McEwen BS. Protective and Damaging Effects of Stress Mediators. New England Journal of Medicine. 1998;338(3):171–9. 10.1056/NEJM199801153380307 . 9428819

[28] Shannon M, King TL, Kennedy HP. Allostasis: a theoretical framework for understanding and evaluating perinatal health outcomes. J Obstet Gynecol Neonatal Nurs. 2007;36(2):125–34. Epub 2007/03/21. 10.1111/j.1552-6909.2007.00126.x . 17371513

[29] Sampson RJ, Raudenbush SW, Earls F. Neighborhoods and Violent Crime: A Multilevel Study of Collective Efficacy. Science. 1997;277(5328):918 10.1126/science.277.5328.918 9252316

[30] Schulz AJ, Kannan S, Dvonch JT, Israel BA, Allen A 3rd, James SA, et al Social and physical environments and disparities in risk for cardiovascular disease: the healthy environments partnership conceptual model. Environ Health Perspect. 2005;113(12):1817–25. Epub 2005/12/07. 10.1289/ehp.7913 16330371 PMC1314928

[31] Cohen S, Kamarck T, Mermelstein R. A global measure of perceived stress. J Health Soc Behav. 1983;24(4):385–96. Epub 1983/12/01. . 6668417

[32] Radloff LS. The CES-D Scale: A Self-Report Depression Scale for Research in the General Population. Applied Psychological Measurement. 1977;1(3):385–401.

[33] Singh-Manoux A, Adler NE, Marmot MG. Subjective social status: its determinants and its association with measures of ill-health in the Whitehall II study. Soc Sci Med. 2003;56(6):1321–33. Epub 2003/02/26. 10.1016/s0277-9536(02)00131-4 . 12600368

[34] Rosseel Y. lavaan: An R Package for Structural Equation Modeling. 2012. 2012;48(2):36 Epub 2012-05-24. 10.18637/jss.v048.i02

[35] Enders CK. The impact of nonnormality on full information maximum-likelihood estimation for structural equation models with missing data. Psychol Methods. 2001;6(4):352–70. Epub 2002/01/10. . 11778677

[36] Lt Hu, Bentler PM. Cutoff criteria for fit indexes in covariance structure analysis: Conventional criteria versus new alternatives. Structural Equation Modeling: A Multidisciplinary Journal. 1999;6(1):1–55. 10.1080/10705519909540118

[37] Stepanikova I, Kukla L. Is Perceived Discrimination in Pregnancy Prospectively Linked to Postpartum Depression? Exploring the Role of Education. Matern Child Health J. 2017;21(8):1669–77. Epub 2017/01/25. 10.1007/s10995-016-2259-7 28116534 PMC5515992

[38] Hall KS, Kusunoki Y, Gatny H, Barber J. Social discrimination, stress, and risk of unintended pregnancy among young women. J Adolesc Health. 2015;56(3):330–7. Epub 2015/01/15. 10.1016/j.jadohealth.2014.11.008 25586228 PMC4339533

[39] van de Loo KFE, Vlenterie R, Nikkels SJ, Merkus P, Roukema J, Verhaak CM, et al Depression and anxiety during pregnancy: The influence of maternal characteristics. Birth. 2018;45(4):478–89. Epub 2018/03/09. 10.1111/birt.12343 . 29517137

[40] Laraia B, Vinikoor-Imler LC, Siega-Riz AM. Food insecurity during pregnancy leads to stress, disordered eating, and greater postpartum weight among overweight women. Obesity (Silver Spring). 2015;23(6):1303–11. Epub 2015/05/12. 10.1002/oby.21075 25959858 PMC6563905

[41] Abrahams Z, Lund C, Field S, Honikman S. Factors associated with household food insecurity and depression in pregnant South African women from a low socio-economic setting: a cross-sectional study. Soc Psychiatry Psychiatr Epidemiol. 2018;53(4):363–72. Epub 2018/02/16. 10.1007/s00127-018-1497-y 29445850 PMC5862931

[42] Giurgescu C, Misra DP, Sealy-Jefferson S, Caldwell CH, Templin TN, Slaughter-Acey JC, et al The impact of neighborhood quality, perceived stress, and social support on depressive symptoms during pregnancy in African American women. Soc Sci Med. 2015;130:172–80. Epub 2015/02/24. 10.1016/j.socscimed.2015.02.006 25703670 PMC4431774

[43] Sealy-Jefferson S, Giurgescu C, Slaughter-Acey J, Caldwell C, Misra D. Neighborhood Context and Preterm Delivery among African American Women: the Mediating Role of Psychosocial Factors. J Urban Health. 2016;93(6):984–96. Epub 2016/10/06. 10.1007/s11524-016-0083-4 27704384 PMC5126020

[44] Dunn N, Inskip H, Kendrick T, Oestmann A, Barnett J, Godfrey K, et al Does perceived financial strain predict depression among young women? Longitudinal findings from the Southampton Women’s Survey. Ment Health Fam Med. 2008;5(1):15–21. Epub 2008/03/01. 22477842 PMC2777551

[45] Goossens J, Van Den Branden Y, Van der Sluys L, Delbaere I, Van Hecke A, Verhaeghe S, et al The prevalence of unplanned pregnancy ending in birth, associated factors, and health outcomes. Hum Reprod. 2016;31(12):2821–33. Epub 2016/11/01. 10.1093/humrep/dew266 . 27798048

