# Supplementary material for: Relationships between psychosocial stressors among pregnant women in San Francisco: A path analysis
Source: PLoS One. 2020 Jun 12;15(6):e0234579. doi: 10.1371/journal.pone.0234579 (PMC7292353; doi:10.1371/journal.pone.0234579)
Supplement: S1 File — (PDF) [file pone.0234579.s001.pdf]

Questionnaire Section A Demographics

Thank you for taking time to participate in this interview.

This interview takes about an hour to complete. I will be asking you questions about your home, job, pregnancy, activities, education, and background. I will also ask you questions about your neighborhood, community, family support, job and working conditions, and your personal views on your exposures to stress. We will not ask you the name of where you work or about your immigration status. We can skip any questions that you would prefer not to answer. You can also stop this interview at any time. Are you ready to begin?

Section A - General Information

Date of questionnaire: ______________________

A.1. What is your birth date?
(If don't know or refused enter question A.1.a)

____
(MM-DD-YY)

A.1.a. Birth date Don't know, Refused, or Missing

- ◯ DK
- ◯ Refused
- ◯ Missing

A.2. What is the last grade of school that you finished?

- ◯ Less than high school
- ◯ High school diploma or GED
- ◯ Some college course work for credit or AA degree
- ◯ Bachelor's degree (4 years)
- ◯ Master's degree
- ◯ Doctoral degree, Ph.D., M.D., or D.D.S
- ◯ DK
- ◯ Ref
- ◯ Missing

A.2.a. If less than HS, how many years of education did you have?

if don't know, enter 97
if refused, enter 98
if missing, enter 99

____
(years)

A.3. I am going to read a list of different racial groups. At the end, please tell me which group you would put yourself in. You can only pick one group.

(Choose only one)

- ◯ Asian
- ◯ Pacific Islander
- ◯ African American/ Black
- ◯ Caucasian/ White
- ◯ Native American
- ◯ Other? (Specify)
- ◯ Don't know
- ◯ Ref
- ◯ Missing

(choose only one)

Please tell me which racial group you would put your baby's father in. You can only pick one group. (Choose only one)	◯ Asian<br>◯ Pacific Islander<br>◯ African American/ Black<br>◯ Caucasian/ White<br>◯ Native American<br>◯ Other? (Specify)<br>◯ Don't know<br>◯ Ref<br>◯ Missing<br>(choose only one)
A.3.a. How many years have you lived in the U.S.?	______________<br>(years)
A.3.b. How many years have you lived in the California?	______________<br>(years)
If Other race, please specify	______________
Is your baby's father of Latino or Hispanic descent, that is: is he Mexican, Mexican American, Chicano, Puerto Rican, Cuban, Central American, South American, some other Latino/Hispanic origin, or any combination of these?	◯ No<br>◯ Yes<br>◯ Don't know<br>◯ Ref<br>◯ Missing
Which of the following represents your baby's father's country of origin?	◯ Mexico<br>◯ El Salvador<br>◯ Other (specify)<br>◯ Don't Know<br>◯ Ref<br>◯ Missing
If other country of origin, please specify	______________
A.4. Are you of Latino or Hispanic descent, that is: are you Mexican, Mexican American, Chicana, Puerto Rican, Cuban, Central American, South American, some other Latina/Hispanic origin, or any combination of these?	◯ No<br>◯ Yes<br>◯ Don't know<br>◯ Ref<br>◯ Missing
A.4.a. Which of the following represents your family's country of origin?	◯ Mexico<br>◯ El Salvador<br>◯ Other (specify)<br>◯ Don't Know<br>◯ Ref<br>◯ Missing
If other country of origin, please specify	______________
Were you born in the Unites States?	◯ No<br>◯ Yes<br>◯ Don't know<br>◯ Refuse<br>◯ Missing

A.5. What is your CURRENT marital status?	◯ Married<br>◯ Widowed<br>◯ Separated<br>◯ Divorced<br>◯ Never married<br>◯ DK<br>◯ Ref<br>◯ Missing
A.6. Although you are not LEGALLY married, are you currently living with a partner as though you are married? For example, with a domestic partner or a common law spouse?	◯ No<br>◯ Yes<br>◯ Don't know<br>◯ Ref<br>◯ Missing
A.7. Are you CURRENTLY in a relationship?	◯ No<br>◯ Yes<br>◯ Don't know<br>◯ Ref<br>◯ Missing
A.8. What is your current spouse/ partner's employment status?	◯ Unemployed/Retired<br>◯ Full-time<br>◯ Part-time<br>◯ Don't Know<br>◯ Ref<br>◯ Missing
A.9. What is the last grade of school that your spouse/partner finished?	◯ Less than high school<br>◯ High school diploma or GED<br>◯ Some college course work for credit or AA degree<br>◯ Bachelor's degree (4 years)<br>◯ Master's degree<br>◯ Doctoral degree, Ph.D., M.D., or D.D.S<br>◯ Don't know<br>◯ Ref<br>◯ Missing
A.9.a. If less than HS, how many years of education did your spouse/partner have?<br><br>if don't know, enter 97<br>if refused, enter 98<br>if missing, enter 99	________________________<br>(years)
A.10. How many children do you have, including adopted or step children?<br><br>If don't know, enter 97<br>If refused, enter 98<br>if missing, enter 99	________________________<br>(number)
A.11. How many of these children live with you?	________________________<br>(number)

Section A - Occupation and income

The next questions are about your occupation and income.

A.12. Thinking back over the PAST 12 MONTHS, how many months did you work for pay at least 15 hours a week?

(Enter upper limit or round up based on participant's answer)

(if never, don't know, refused, or missing, enter question A.12.a.)

____
(months)

A.12.a. Working for pay: Never, Don't know, Refused, or Missing

◯ Never
◯ Don't know
◯ Ref
◯ Missing

A.13. On average how many hours per week did you work for pay during the PAST 12 MONTHS?

(Enter upper limit or round up based on participant's answer)

If don't know, enter 97
If Refused, enter 98
If Missing, enter 99

____
(hours)

A.14. When was the last time you were employed?

____
(MM-DD-YY)

A.15. Are you currently covered under a health insurance or managed care program?

◯ No
◯ Yes
◯ Don't know
◯ Ref
◯ Missing

A.15.a. Is your health insurance Medi-Cal?

◯ No
◯ Yes
◯ Don't know
◯ Ref
◯ Missing

A.16. Which letter on this card best represents the combined income of everyone in your HOUSEHOLD, before taxes?<br><br>(Show Card A1)<br><br>If respondent doesn't know, probe: Please give me your best guess. If Question A.16 answered directly, skip to question A.20. Otherwise, probe income using questions A.17 - A.19	◯ A. Less than $5,000<br>◯ B. $5,000 - 9,999<br>◯ C. $10,000 - 14,999<br>◯ D. $15,000-19,999<br>◯ E $20,000-24,999<br>◯ F $25,000-29,999<br>◯ G $30,000-34,999<br>◯ H $35,000-39,999<br>◯ I $40,000-44,999<br>◯ J $45,000-49,999<br>◯ K $50,000-59,999<br>◯ L $60,000-69,999<br>◯ M $70,000-79,999<br>◯ N $80,000-89,999<br>◯ O $90,000-99,999<br>◯ P $100,000-124,999<br>◯ Q $125,000-149,999<br>◯ R $150,000-174,999<br>◯ S $175,000-199,999<br>◯ T $200,000 and over<br>◯ Don't know<br>◯ Ref<br>◯ Missing
A.17. You may not be able to tell me exactly what your total household income was, but would you say that it is more or less than $20,000?	◯ Less than $20,000<br>◯ More than $20,000<br>◯ Don't know<br>◯ Ref<br>◯ Missing
A.18. Is it more or less than $40,000?	◯ Less than $40,000<br>◯ More than $40,000<br>◯ Don't know<br>◯ Ref<br>◯ Missing
A.19. Is it more or less than $80,000?	◯ Less than $80,000<br>◯ More than $80,000<br>◯ Don't know<br>◯ Ref<br>◯ Missing

A.20. Now I'd like to talk about assets--that is, things that you or other members of your household could sell, if you wanted, to earn additional money. I will read a list of assets. Please tell me if you and your spouse/partner own any of these types of assets.

(Check all that apply)

	No	Yes	Don't know	Ref	Missing
A.20.a. Real estate other than your main home, such as a second home or vacation home, land, rental property, a property partnership, or money owed to you on a land contract or mortgage	◯	◯	◯	◯	◯
A.20.b. Part or all of a business or farm	◯	◯	◯	◯	◯
Money or assets that are held in retirement accounts under your or your (spouse/partner's or another household member's) name, such as a pension, an annuity, an IRA, KEOGH or a 401(k) account	◯	◯	◯	◯	◯
A.20.d. Savings or investments, such as shares of stock, mutual funds, bonds, bond funds, or treasury bills, checking or savings accounts, certificates of deposit, or money market funds	◯	◯	◯	◯	◯
A.20.e. One or more working cars (not a leased car)	◯	◯	◯	◯	◯

A.21. Now, thinking about all the things you've mentioned above, including __________________ [Fill From Above], and not including your main home, how much would you say that all these assets are worth together? Again, this does not include your main home.

(Can use Card A1 to assist)

◯ Less than $500
◯ $500 - 4,999
◯ $5,000 - 9,999
◯ $10,000 - 24,999
◯ $25,000 - 49,999
◯ $50,000 - 99,999
◯ $100,000 - 199,999
◯ $200,000 - 499,999
◯ $500,000 or more
◯ Don't Know
◯ Ref
◯ Missing

A.22. Now, please tell me whether the home where you live is:	◯ Owned or being bought by you and/or your spouse/partner<br>◯ Rented for money<br>◯ Occupied without payment of money or rent<br>◯ Something else (specify)<br>◯ Don't Know<br>◯ Ref<br>◯ Missing
Home living status specify	__________________________________
A.23. How hard is it for you to pay for very basics like food, housing, medical care, and heating? Would you say not difficult at all, not very difficult, somewhat difficult, or very difficult?	◯ Not difficult at all<br>◯ Not very difficult<br>◯ Somewhat difficult<br>◯ Very difficult<br>◯ Don't Know<br>◯ Ref<br>◯ Missing

Next, I would like to ask you some questions about the number of people in your household.

Counting yourself, how many people live in your household?

A.24. How many of these people are under 18?<br><br>If don't know, enter 97<br>If refused, enter 98<br>if missing, enter 99	__________________________________<br>(number)
A.25. How many are between 18 and 64?<br><br>If don't know, enter 97<br>If refused, enter 98<br>if missing, enter 99	__________________________________<br>(number)
A.26. How many are 65 or older?<br><br>If don't know, enter 97<br>If refused, enter 98<br>if missing, enter 99	__________________________________<br>(number)

Questionnaire section B - Health

Section B: Pregnancy.

The following questions are about your current pregnancy.

B.1. During the month before you got pregnant, how many times a week did you take a multivitamin or a prenatal vitamin? These are pills that contain many different vitamins and minerals.

- ◯ I didn't take a multivitamin or a prenatal vitamin at all
- ◯ 1 to 3 times a week
- ◯ 4 to 6 times a week
- ◯ Every day of the week
- ◯ Don't Know
- ◯ Ref
- ◯ Missing

B.2. Thinking back to just before you got pregnant, how did you feel about becoming pregnant?

- ◯ I didn't want to be pregnant then
- ◯ I wanted to be pregnant then
- ◯ I wanted to be pregnant later
- ◯ I wanted to be pregnant sooner
- ◯ Don't Know
- ◯ Ref
- ◯ Missing

B. Health (cont.) - Mood

Now I have a few questions about your mood during the past year.

	Rarely	Some of the time	Often	Most of the time	Don't know	Ref	Missing
B.3. In the past year, how often were you bothered by things that usually do not bother you	◯	◯	◯	◯	◯	◯	◯
B.4. In the past year, how often did you have trouble staying focused on what you were doing?	◯	◯	◯	◯	◯	◯	◯
B.5. In the past year, how often did you feel depressed?	◯	◯	◯	◯	◯	◯	◯
B.6. In the past year, how often did you feel that everything you did was an effort?	◯	◯	◯	◯	◯	◯	◯
B.7. In the past year, how often did you feel hopeful about the future?	◯	◯	◯	◯	◯	◯	◯
B.8. In the past year, how often did you feel fearful?	◯	◯	◯	◯	◯	◯	◯
B.9. In the past year, how often was your sleep restless?	◯	◯	◯	◯	◯	◯	◯

B.10. In the past year, how often were you happy?	◯	◯	◯	◯	◯	◯	◯
B.11. In the past year, how often did you feel lonely?	◯	◯	◯	◯	◯	◯	◯
B.12. In the past year, how often did you feel that you could get going?	◯	◯	◯	◯	◯	◯	◯

B. Health (cont.) - Smoking and Drinking

My next few questions about smoking and drinking.

B.13. Do you smoke?

◯ No
◯ Yes
◯ Don't Know
◯ Ref
◯ Missing

B.14. How many cigarettes per day do you usually smoke?

(1 pack = 20 cigarettes)

If Don't know, enter 97
If Refused, enter 98
If Missing, enter 99

____
(# cigs/day)

B.15. Did you smoke today?

◯ No
◯ Yes
◯ Don't Know
◯ Ref
◯ Missing

B.16. How long ago did you last smoke? (enter number)

Enter unit in next question.
If Never smoked, enter next question.
If don't know, refused, or missing, enter next question.

____
(number)

B.16.a. How long ago did you last smoke? (enter unit)

◯ Never
◯ Minutes
◯ Hours
◯ Months
◯ Years
◯ Don't know
◯ Ref
◯ Missing
(time unit)

B.17. During the last 12 months, how often did you usually have any kind of drink containing alcohol? By a drink we mean something like a 12 ounce can or glass of beer or cooler, a 5 ounce glass of wine, or a drink containing 1 shot of liquor.	◯ I never drank any alcohol in my life<br>◯ I did not drink any alcohol in the past year, but I did drink in the past<br>◯ 1 or 2 times in the past year<br>◯ 3 to 11 times in the past year<br>◯ Once a month<br>◯ 2 to 3 times a month<br>◯ Once a week<br>◯ Twice a week<br>◯ 3 to 4 times a week<br>◯ 5 to 6 times a week<br>◯ Every day<br>◯ Don't Know<br>◯ Ref<br>◯ Missing
B.18. Did you have an alcoholic drink today?	◯ No<br>◯ Yes<br>◯ Don't Know<br>◯ Ref<br>◯ Missing
B.19. How long ago did you last have an alcoholic drink? (enter number)<br><br>Enter unit in next question.<br>If Never smoked, enter next question.<br>If don't know, refused, or missing, enter next question.	__________________________________<br>(number)
B.19.a. How long ago did you last have an alcoholic drink? (enter unit)	◯ Never<br>◯ Minutes<br>◯ Hours<br>◯ Days<br>◯ Months<br>◯ Years<br>◯ Don't know<br>◯ Ref<br>◯ Missing<br>(time unit)

Questionnaire section C - Stress

The first questions are about your current job or most recent job, if you've had one. We are specifically interested in the job where you worked the most hours in a given week

C - Stress: Work

C.1. Are you currently working for pay, looking for work, retired, a homemaker or raising children full time, a student, or something else?

- ◯ Working for pay
- ◯ Looking for work
- ◯ Retired
- ◯ Homemaker or raising children full time
- ◯ Student
- ◯ Something else
- ◯ Don't Know
- ◯ Ref
- ◯ Missing

If 'something else', please explain: ______________________

C.2. How many jobs do you have?

If Don't know, enter 97
If Refused, enter 98
If Missing, enter 99

______________________ (number)

C.3.a. What is/are your current jobs? (Job 1) ______________________

C.3.a.1. How many hours per week do you work at your job?

If Don't know, enter 97
If Refused, enter 98
If Missing, enter 99

______________________ (hours)

C.3.b. What is/are your current second jobs? (Job 2) ______________________

C.3.b.1. How many hours per week do you work at your second job? (job 2)

If Don't know, enter 97
If Refused, enter 98
If Missing, enter 99

______________________ (hours)

C.4. What was your most recent job?
If never worked, don't know, refused, or missing, skip to Stress-Life Events section.

____

C.4.a. How long ago did you have this job? (how many months)

______________________ (months)

C.5. How long have you worked in your (first) current job [OR] How long did you work in your most recent job?<br><br>Enter unit in next question.<br>If Never worked, enter next question.<br>If don't know, refused, or missing, enter next question.	______________________<br>(number)
C.5. How long have you worked in your (first) current job [OR] How long did you work in your most recent job?<br><br>Enter unit	◯ Never worked<br>◯ months<br>◯ years<br>◯ Don't know<br>◯ Ref<br>◯ Missing<br>(units)
C.5.a. How long have you worked in your SECOND current job?<br><br>Enter unit in next question.<br>If Never worked, enter next question.<br>If don't know, refused, or missing, enter next question.	______________________<br>(number)
C.5.a. How long have you worked in your SECOND current job?<br><br>Enter unit	◯ No second job<br>◯ months<br>◯ years<br>◯ Don't know<br>◯ Ref<br>◯ Missing<br>(unit)
Since becoming pregnant, have you performed a job or activity that involved touching or handling receipts from an ATM machine, cash register's machine, store, or credit card machine?	◯ Yes<br>◯ No
In what week of your pregnancy did you start this job/activity?<br>[If before current pregnancy, write 'preconception' in field.]	______________________
In what week of your pregnancy did you end this job/activity?<br>[If ongoing, write 'ongoing' in field.]	______________________
What type of job or activity is/was this?	____________________________
When performing this job or activity, how many hours per week would you say you come into contact with a receipt?	______________________<br>(Hours/Week)

Stress - Main job (job 1)

I will read a list of things that people sometimes tell us about their work. Thinking about your main current job, or your most recent job, please tell me whether you strongly disagree, somewhat disagree, neither agree nor disagree, somewhat agree, or strongly agree with each statement.

	strongly disagree	somewhat disagree	neither agree nor disagree	somewhat agree	strongly agree	Don't know	Ref	Missing
C.6. My job allows me to make a lot of decisions on my own.	○	○	○	○	○	○	○	○
C.7. I have an opportunity to develop my own special abilities.	○	○	○	○	○	○	○	○
C.8. I am not asked to do an excessive amount of work.	○	○	○	○	○	○	○	○
C.9. My job leaves me feeling too tired and stressed after work.	○	○	○	○	○	○	○	○
C.10. Considering my efforts and achievements, my salary (pay) is fair.	○	○	○	○	○	○	○	○

Stress - Second job (job 2)

I am going to repeat each of the statements again. This time please think about your second job and tell me whether you strongly disagree, somewhat disagree, neither agree nor disagree, somewhat agree, or strongly agree with each statement.

	strongly disagree	somewhat disagree	neither agree nor disagree	somewhat agree	strongly agree	Don't know	Ref	Missing
C.11. My job allows me to make a lot of decisions on my own.	○	○	○	○	○	○	○	○
C.12. I have an opportunity to develop my own special abilities.	○	○	○	○	○	○	○	○
C.13. I am not asked to do an excessive amount of work.	○	○	○	○	○	○	○	○
C.14. My job leaves me feeling too tired and stressed after work.	○	○	○	○	○	○	○	○
C.15. Considering my efforts and achievements, my salary (pay) is fair.	○	○	○	○	○	○	○	○

C - Stress (cont.): Life Events

Next, I'd like to ask some questions about how things have been going for you during the last five years. I'll read several statements, and after each one, I'd like you to tell me how often during the last five years you felt that way. Your choices for answers are: never, rarely, sometimes, often, or very often.

	Never	Rarely	Sometimes	Often	Very often	Don't know	Ref	Missing
C.16. How often in the past 5 years have you felt that you were unable to control the important things in your life?	○	○	○	○	○	○	○	○
C.17. How often in the past 5 years have you felt confident about your ability to handle your personal problems?	○	○	○	○	○	○	○	○
C.18. How often in the past 5 years have you felt that things were going your way?	○	○	○	○	○	○	○	○
C.19. How often in the past 5 years have you felt that difficulties were piling up so high that you could not overcome them?	○	○	○	○	○	○	○	○
C.20. How often in the past 5 years did you have problems in relationships with friends or neighbors?	○	○	○	○	○	○	○	○
C.21. How often in the past 5 years were you responsible for the care and well being of a parent or any older relative?	○	○	○	○	○	○	○	○
C.22. How often in the past 5 years were you responsible for the care and well-being of a child who needs or uses more medical care, mental health, or educational services than is usual for most children of the same age?	○	○	○	○	○	○	○	○

C - Stress (cont.): Racism/Discrimination

Earlier I asked you to self-identify your race and ethnicity. Now I will ask you how other people identify you and treat you.

C.23. How do other people usually classify you in this country? Would you say: White, Black or African American, Hispanic or Latino, Asian, Native Hawaiian or Other Pacific Islander, American Indian or Alaska Native, or some other group?

- ◯ White
- ◯ Black or African American
- ◯ Hispanic or Latino
- ◯ Asian
- ◯ Native Hawaiian or Other Pacific Islander
- ◯ American Indian or Alaska Native
- ◯ Some other group (please specify) __________
- ◯ DK
- ◯ Ref
- ◯ Missing

If Other race, please specify

____

Next, I'll read three statements about your experiences with racism. After each one, please tell me how often you experience these things. Your choices for answers are: never, rarely, sometimes, often, or very often.

	Never	Rarely	Sometimes	Often	Very often	Don't know	Ref	Missing
C.24. How often do you think about your race or ethnicity?	◯	◯	◯	◯	◯	◯	◯	◯
C.25. How often do you feel that racial/ethnic groups who are not white, such as African Americans, Asians, or Latinos, are discriminated against?	◯	◯	◯	◯	◯	◯	◯	◯
C.26. How often do you feel that you, personally, have been discriminated against because of your race, ethnicity, ancestry religion, or color?	◯	◯	◯	◯	◯	◯	◯	◯

C.27. When you feel that you have been treated unfairly or discriminated against, do you usually:

- ◯ Accept it as a fact of life
- ◯ Try to do something about it
- ◯ Don't Know
- ◯ Ref
- ◯ Missing

C.28. If you have been treated unfairly or discriminated against, do you usually:

- ◯ Talk to other people about it.
- ◯ Keep it to yourself
- ◯ Don't Know
- ◯ Ref
- ◯ Missing

C - Stress (cont.): Acute Stress

This next set of questions is about things that may have happened during the last 12 months. For each item, say 'Yes' if it happened to you 'No' if it did not. (It may help to use the calendar.)

	No	Yes	NA (not applicable)	Don't know	Ref	Missing
C.29. A close family member was very sick and had to go into the hospital	○	○	○	○	○	○
C.30. I got separated or divorced from my husband or partner	○	○	○	○	○	○
C.31. I moved to a new address	○	○	○	○	○	○
C.32. My spouse or partner lost his/her job	○	○	○	○	○	○
C.33. I lost my job even though I wanted to go on working	○	○	○	○	○	○
C.34. I argued with my spouse or partner more than usual	○	○	○	○	○	○
C.35. My spouse or partner said s/he didn't want me to be pregnant	○	○	○	○	○	○
C.36. I had a lot of bills I couldn't pay	○	○	○	○	○	○
C.37. I was in a physical fight	○	○	○	○	○	○
C.38. My spouse or partner had serious legal problems.	○	○	○	○	○	○
C.39. Someone very close to me had a problem with drinking or drugs	○	○	○	○	○	○
C.40. Someone very close to me died	○	○	○	○	○	○
C.40.a I or a close family member has experienced immigration problems	○	○	○	○	○	○

C - Stress (cont.): Social Support

Now, I'm going to describe some problems that everyone faces now and then. For each of the following, please tell how often you feel you would be able to get help from your family, friends, neighbors and co-workers without having to pay them. Your choices for answers are: never, rarely, sometimes, often, or very often.

	Never	Rarely	Sometimes	Often	Very often	Don't know	Ref	Missing
C.41. If you were sick, how often would there be somebody who could help care for you?	◯	◯	◯	◯	◯	◯	◯	◯
C.42. If you were worried about an important personal matter, how often would there be somebody you could talk to about it?	◯	◯	◯	◯	◯	◯	◯	◯
C.43. How often is there somebody who makes you feel loved and cared for?	◯	◯	◯	◯	◯	◯	◯	◯

C - Stress (cont.): Religiosity

Now I have two questions about your religious beliefs and practices.

C.44. Do you consider yourself a religious person?

◯ No
◯ Yes
◯ Don't know
◯ Ref
◯ Missing

C.45. How often do you attend a religious service at a church, temple, mosque or other place of worship?

◯ Never
◯ At least once a month
◯ Holidays only
◯ Don't know
◯ Ref
◯ Missing

Section C - Stress (cont.): Neighborhood

I would like to get an idea of how you feel about the neighborhood you've lived in the longest during the last 5 years. By neighborhood, I mean the streets surrounding your house and also those that are within walking distance of your home.
I am going to read you a list of statements. For each one, please tell me whether you strongly disagree, somewhat disagree, neither agree nor disagree, somewhat agree or strongly agree.

	strongly disagree	somewhat disagree	neither agree nor disagree	somewhat agree	strongly agree	Don't know	Ref	Missing
C.46. I think this neighborhood is a good place for me to live.	○	○	○	○	○	○	○	○
C.47. People around here are willing to help their neighbors.	○	○	○	○	○	○	○	○
C.48. I feel safe in this neighborhood.	○	○	○	○	○	○	○	○
C.49. People in this neighborhood can be trusted.	○	○	○	○	○	○	○	○
C.50. This is a close-knit neighborhood.	○	○	○	○	○	○	○	○
C.51. There is heavy car or truck traffic in this neighborhood.	○	○	○	○	○	○	○	○
C.52. I would move out of this neighborhood if I could.	○	○	○	○	○	○	○	○
C.53. People in this neighborhood generally don't get along with each other.	○	○	○	○	○	○	○	○
C.54. People in this neighborhood don't share the same values.	○	○	○	○	○	○	○	○
C.55. There is a lot of loud noise from cars, motorcycles, music, neighbors, or airplanes in my neighborhood.	○	○	○	○	○	○	○	○
C.56. My neighborhood has a lot of vacant lots or vacant houses.	○	○	○	○	○	○	○	○

Still thinking of the neighborhood you lived in the longest during the past 5 years, Please tell me how likely it is that your neighbors could be counted on to intervene if:

	Very unlikely	Unlikely	Neither likely nor unlikely	Likely	Very likely	Don't know	Ref	Missing
C.57. Children were skipping school and hanging out on a street corner	○	○	○	○	○	○	○	○
C.58. Children were spray-painting graffiti on a local building	○	○	○	○	○	○	○	○
C.59. Children were showing disrespect to an adult	○	○	○	○	○	○	○	○
C.60. A fight broke out in front of their house	○	○	○	○	○	○	○	○

C.61. How many years and months did you live in the neighborhood that we just discussed?

____ years

Enter month in next questions
if Don't know, enter 97
if Ref, enter 98
if Missing, enter 99

____
(years)

C.61. How many years and months did you live in the neighborhood that we just discussed?

____ months

Enter month in next questions
if Don't know, enter 97
if Ref, enter 98
if Missing, enter 99

____
(months)

C.62. Is the neighborhood that we just discussed your current neighborhood?

○ No
○ Yes
○ Don't know
○ Ref
○ Missing

C.63. In the past 5 years how many neighborhoods have you lived in?

if Don't know, enter 97
if Ref, enter 98
if Missing, enter 99

____
(number)

I am going to read you the same list of statements. Thinking of your current neighborhood, please tell me whether you strongly disagree, somewhat disagree, neither agree nor disagree, somewhat agree or strongly agree.

Branching exists: If neighborhood lived in the longest is also current neighborhood, matrix questions will not show up. Disregard this header.

	Strongly disagree	Somewhat disagree	Neither agree nor disagree	Somewhat agree	Strongly agree	Don't Know	Ref	Missing
C.64. I think my current neighborhood is a good place for me to live.	○	○	○	○	○	○	○	○
C.65. People in my current are willing to help their neighbors.	○	○	○	○	○	○	○	○
C.66. I feel safe in my current neighborhood.	○	○	○	○	○	○	○	○
C.67. People in my current neighborhood can be trusted.	○	○	○	○	○	○	○	○
C.68. This is a close-knit neighborhood.	○	○	○	○	○	○	○	○
C.69. There is heavy car or truck traffic in my current neighborhood.	○	○	○	○	○	○	○	○
C.70. I would move out of my current neighborhood if I could.	○	○	○	○	○	○	○	○
C.71. People in my current neighborhood generally don't get along with each other.	○	○	○	○	○	○	○	○
C.72. People in my current neighborhood don't share the same values.	○	○	○	○	○	○	○	○
C.73. There is a lot of loud noise from cars, motorcycles, music, neighbors, or airplanes in my current neighborhood.	○	○	○	○	○	○	○	○
C.74. My current neighborhood has a lot of vacant lots or vacant houses.	○	○	○	○	○	○	○	○

Now I'm going to ask you how likely it is that your current neighbors could be counted on to intervene if:

Branching exists: If neighborhood lived in the longest is also current neighborhood, matrix questions will not show up. Disregard this header.

	Very Unlikely	Unlikely	Neither likely nor unlikely	likely	Very likely	Don't Know	Ref	Missing
C.75. Children were skipping school and hanging out on a street corner	○	○	○	○	○	○	○	○
C.76. Children were spray-painting graffiti on a local building	○	○	○	○	○	○	○	○
C.77. Children were showing disrespect to an adult	○	○	○	○	○	○	○	○
C.78. A fight broke out in front of their house	○	○	○	○	○	○	○	○

C.79. During the past 5 years, how many times have you moved from one house or apartment to another?

____
(number of moves)

if Don't know, enter 97
if Ref, enter 98
if Missing, enter 99

SHOW CARD B.1.

Next, please look at this ladder. Think of this ladder as representing where people stand in their COMMUNITIES [Neighborhood] TODAY.

C.80. People define their communities in different ways; please think of your community in the way that is most meaningful to you. A "1" represents people who have the lowest standing in your community, a "10" represents people who have the highest standing in your community. Where would you place yourself today, on this scale? You may choose any number between 1 and 10.

____

Section C - Stress (cont.): Food Security

These next questions are about the food eaten in your household in the last 12 months, since __________[prefill name of current month] of last year, and whether you were able to afford the food you needed in that time.

C.81. Which of these statements best describes the food situation in your household in the last 12 months:

- ◯ (I / We) often could not afford enough to eat
- ◯ (I / We) sometimes could not afford enough to eat
- ◯ (I / We) could always afford enough to eat but not always the food we should eat
- ◯ (I / We) could always afford to eat good nutritious meals
- ◯ Don't Know
- ◯ Ref
- ◯ Missing

C.81.a. I am going to list some reasons why people don't always have enough to eat. Please tell me if each reason is the reason why YOU don't always have enough to eat. You can say "yes" to more than one reason.

	No	Yes	NA	Don't Know	Ref	Missing
C.81.a.1. Not enough money for food	◯	◯	◯	◯	◯	◯
C.81.a.2. Not enough time for shopping or cooking	◯	◯	◯	◯	◯	◯
C.81.a.3. Too hard to get to the store	◯	◯	◯	◯	◯	◯
C.81.a.4. On a diet	◯	◯	◯	◯	◯	◯
C.81.a.5. No working stove available	◯	◯	◯	◯	◯	◯
C.81.a.6. Not able to cook or eat because of health problems	◯	◯	◯	◯	◯	◯

C.81.b. I am going to list some reasons why people don't always have the quality or variety of food they want. Please tell me if each reason is the reason why YOU don't always have the kinds of food you want to eat. You can say "yes" to more than one reason.

	No	Yes	NA	Don't know	Ref	Missing
C.81.b.1. Not enough money for the quality or variety of food I would like	◯	◯	◯	◯	◯	◯
C.81.b.2. The quality or kinds of food (I/we) want are not available at the store I regularly shop at	◯	◯	◯	◯	◯	◯
C.81.b.3. Not enough time for shopping or cooking	◯	◯	◯	◯	◯	◯

C.81.b.4. Too hard to get to the store	◯	◯	◯	◯	◯	◯
C.81.b.5. On a special diet	◯	◯	◯	◯	◯	◯

Now I'm going to read you several statements that people have made about their food situation. For each statement, please tell me whether the statement was true for your household in the last 12 months. Your choices for answers are: never true, sometimes true, or often true.

C.82. "The food that we bought just didn't last, and we didn't have money to get more." Was this often, sometimes, or never true for your household in the last 12 months?

◯ Never True
◯ Sometimes True
◯ Often True
◯ Don't Know
◯ Ref
◯ Missing

C.83. "We couldn't afford to eat balanced and nutritious meals." Was this never, sometimes, or often true for your household?

◯ Never True
◯ Sometimes True
◯ Often True
◯ Don't know
◯ Ref
◯ Missing

C.84. In the last 12 months, since last ___________[prefill name of current month], did you or other adults in your household ever cut the size of your meals or skip meals because there wasn't enough money for food?

◯ No
◯ Yes
◯ Don't know
◯ Ref
◯ Missing

C.85. Did this happen almost every month, some months but not every month, or in only 1 or 2 months?

◯ Only for 1 or 2 months
◯ Some months but not every month
◯ Almost every month
◯ Don't Know
◯ Ref
◯ Missing

C.86. In the last 12 months, did you ever eat less than you felt you should because there wasn't enough money to buy food?

◯ No
◯ Yes
◯ Don't know
◯ Ref
◯ Missing

C.87. In the last 12 months, were you ever hungry but didn't eat because you couldn't afford more food?

◯ No
◯ Yes
◯ Don't know
◯ Ref
◯ Missing

C.88. In the past year have you received any SNAP benefits or food stamps?

◯ No
◯ Yes
◯ Don't know
◯ Ref
◯ Missing

C.88.a. Are you currently receiving food stamps?	◯ No<br>◯ Yes<br>◯ Don't know<br>◯ Ref<br>◯ Missing
C.89. How often do you eat fast-food or take-out food?	◯ Less than once a month<br>◯ 1-3 times a month<br>◯ Once a week<br>◯ Every other day<br>◯ 4-6 times a week<br>◯ Once a day<br>◯ More than once a day<br>◯ Don't Know<br>◯ Ref<br>◯ Missing
C.90. How often do you eat meals that you or someone else prepares at home?	◯ Less than once a month<br>◯ 1-3 times a month<br>◯ Once a week<br>◯ Every other day<br>◯ 4-6 times a week<br>◯ Once a day<br>◯ More than once a day<br>◯ Don't Know<br>◯ Ref<br>◯ Missing

Questionnaire section D - Personal Care Products

D.1. Please tell me if you use any of these products every day. [Mark all the apply]	☐ Shampoo, Conditioner<br>☐ Make up<br>☐ Hair gel or Hair spray<br>☐ Lip balm or moisturizers<br>☐ Lotion<br>☐ Sun screen<br>☐ Deodorant<br>☐ Vaginal wash<br>☐ Fragrance, Body spray or Perfume<br>☐ Nail polish or Nail polish remover<br>☐ Colgate Total toothpaste
D.2. Did you use any of these products today? [Mark all the apply]	☐ Shampoo, Conditioner<br>☐ Make up<br>☐ Hair gel or Hair spray<br>☐ Lip balm or moisturizers<br>☐ Lotion<br>☐ Sun screen<br>☐ Deodorant<br>☐ Vaginal wash<br>☐ Fragrance, Body spray or Perfume<br>☐ Nail polish or Nail polish remover<br>☐ Colgate Total toothpaste
D.3. I am going to read a list of household cleaning products. Please tell me if you use any of these products every day.	☐ Bleach<br>☐ Air freshener plug ins or sprays<br>☐ Ammonia<br>☐ Scented candles or incense<br>☐ Cleaning solvents (stain removers, degreasers, and oven cleaners)<br>☐ Cleaning sprays (ex: windows)<br>☐ Floor or furniture polish or wax
D.4. Did you use any of these products today?	☐ Bleach<br>☐ Air freshener plug ins or sprays<br>☐ Ammonia<br>☐ Scented candles or incense<br>☐ Cleaning solvents (stain removers, degreasers, and oven cleaners)<br>☐ Cleaning sprays (ex: windows)<br>☐ Floor or furniture polish or wax

Questionnaire section E - PBDE & PFC Exposure

I am now going to ask you about the foods you have eaten since you became pregnant.
I am going to ask you about many foods. Please tell me how many times a day, week, month or year you eat each food item.

Ask this way: How many times a day, week, month or year to you eat [ITEM]?

1. Red meat (for example, beef, pork, lamb or goat)
Enter number.

Enter unit in next question.
If Don't know, Refused, or Missing, enter in next question

____
(# times)

---

1. Red meat (for example, beef, pork, lamb or goat)

Enter unit

◯ Day
◯ Week
◯ Month
◯ Year
◯ Never
◯ Don't know
◯ Ref
◯ Missing
(frequency unit)

---

2. Poultry (for example, chicken, turkey or duck)

Enter number

Enter unit in next question.
If Don't know, Refused, or Missing, enter in next question

____
(# times)

---

2. Poultry (for example, chicken, turkey or duck)

Enter unit

◯ Day
◯ Week
◯ Month
◯ Year
◯ Never
◯ Don't know
◯ Ref
◯ Missing
(frequency unit)

---

3. Liver, liver pate, liver wurst or any other kind of liver dish

Enter number

Enter unit in next question.
If Don't know, Refused, or Missing, enter in next question

____
(# times)

3. Liver, liver pate, liver wurst or any other kind of liver dish<br><br>Enter unit	◯ Day<br>◯ Week<br>◯ Month<br>◯ Year<br>◯ Never<br>◯ Don't know<br>◯ Ref<br>◯ Missing<br>(frequency unit)
4. Fish from stores, markets or restaurants. This includes any fish that is fresh, frozen, smoked, dried, or canned, such as canned tuna or sardines.<br><br>Enter number.<br><br>Enter unit in next question.<br>If Don't know, Refused, or Missing, enter in next question	________________________<br>(# times)
4. Fish from stores, markets or restaurants. This includes any fish that is fresh, frozen, smoked, dried, or canned, such as canned tuna or sardines.<br><br>Enter unit	◯ Day<br>◯ Week<br>◯ Month<br>◯ Year<br>◯ Never<br>◯ Don't know<br>◯ Ref<br>◯ Missing<br>(frequency unit)
5. Fish caught by you or someone you know. Please do not include fish that came from stores, markets, or restaurants.<br><br>Enter number.<br><br>Enter unit in next question.<br>If Don't know, Refused, or Missing, enter in next question	________________________<br>(# times)
5. Fish caught by you or someone you know. Please do not include fish that came from stores, markets, or restaurants.<br><br>Enter unit	◯ Day<br>◯ Week<br>◯ Month<br>◯ Year<br>◯ Never<br>◯ Don't know<br>◯ Ref<br>◯ Missing<br>(frequency unit)
6. Shellfish from stores, markets or restaurants. This includes any shellfish, like shrimp, that is fresh, frozen, smoked, dried, or canned, such as canned clams or mussels.<br><br>Enter number.<br><br>Enter unit in next question.<br>If Don't know, Refused, or Missing, enter in next question	________________________<br>(# times)

6. Shellfish from stores, markets or restaurants. This includes any shellfish, like shrimp, that is fresh, frozen, smoked, dried, or canned, such as canned clams or mussels.<br><br>Enter unit	◯ Day<br>◯ Week<br>◯ Month<br>◯ Year<br>◯ Never<br>◯ Don't know<br>◯ Ref<br>◯ Missing<br>(frequency unit)
7. Shellfish caught by you or someone you know. Please do not include shellfish that came from stores, markets, or restaurants.<br><br>Enter number.<br><br>Enter unit in next question.<br>If Don't know, Refused, or Missing, enter in next question	____________________<br>(# times)
7. Shellfish caught by you or someone you know. Please do not include shellfish that came from stores, markets, or restaurants.<br><br>Enter unit	◯ Day<br>◯ Week<br>◯ Month<br>◯ Year<br>◯ Never<br>◯ Don't know<br>◯ Ref<br>◯ Missing<br>(frequency unit)
8. Eggs<br><br>Enter number.<br><br>Enter unit in next question.<br>If Don't know, Refused, or Missing, enter in next question	____________________<br>(# times)
8. Eggs<br><br>Enter unit	◯ Day<br>◯ Week<br>◯ Month<br>◯ Year<br>◯ Never<br>◯ Don't know<br>◯ Ref<br>◯ Missing<br>(frequency unit)
9. Soy milk<br><br>Enter number.<br><br>Enter unit in next question.<br>If Don't know, Refused, or Missing, enter in next question	____________________<br>(# times)

9. Soy milk

Enter unit

◯ Day
◯ Week
◯ Month
◯ Year
◯ Never
◯ Don't know
◯ Ref
◯ Missing
(frequency unit)

9. Milk

Enter number.

Enter unit in next question.
If Don't know, Refused, or Missing, enter in next question

____
(# times)

9. Milk

Enter unit

◯ Day
◯ Week
◯ Month
◯ Year
◯ Never
◯ Don't know
◯ Ref
◯ Missing
(frequency unit)

11. What kind of milk?

◯ Whole (4%)
◯ 2% Low-fat
◯ 1% Low-fat
◯ Non-fat
◯ Never
◯ DK
◯ Ref
◯ Missing

12. Cheese

Enter number.

Enter unit in next question.
If Don't know, Refused, or Missing, enter in next question

____
(# times)

12. Cheese

Enter unit

◯ Day
◯ Week
◯ Month
◯ Year
◯ Never
◯ Don't know
◯ Ref
◯ Missing
(frequency unit)

13. Yogurt (including frozen yogurt)

Enter number.

Enter unit in next question.
If Don't know, Refused, or Missing, enter in next question

__________________ (# times)

13. Yogurt (including frozen yogurt)

Enter unit

- ◯ Day
- ◯ Week
- ◯ Month
- ◯ Year
- ◯ Never
- ◯ Don't know
- ◯ Ref
- ◯ Missing

(frequency unit)

14. What kind of yogurt?

- ◯ Whole (4%)
- ◯ 2% Low-fat
- ◯ 1% Low-fat
- ◯ Non-fat
- ◯ Never
- ◯ DK
- ◯ Ref
- ◯ Missing

15. Ice Cream (not including popsicles)

Enter number.

Enter unit in next question.
If Don't know, Refused, or Missing, enter in next question

__________________ (# times)

15. Ice Cream (not including popsicles)

Enter unit

- ◯ Day
- ◯ Week
- ◯ Month
- ◯ Year
- ◯ Never
- ◯ Don't know
- ◯ Ref
- ◯ Missing

(frequency unit)

16. Butter (either on food or for cooking or baking)

Enter number.

Enter unit in next question.
If Don't know, Refused, or Missing, enter in next question

__________________ (# times)

16. Butter (either on food or for cooking or baking)<br><br>Enter unit	◯ Day<br>◯ Week<br>◯ Month<br>◯ Year<br>◯ Never<br>◯ Don't know<br>◯ Ref<br>◯ Missing<br>(frequency unit)
17. Lard (either for cooking or on food)<br>Enter number.<br><br>Enter unit in next question.<br>If Don't know, Refused, or Missing, enter in next question	______________________<br>(# times)
17. Lard (either for cooking or on food)<br><br>Enter unit	◯ Day<br>◯ Week<br>◯ Month<br>◯ Year<br>◯ Never<br>◯ Don't know<br>◯ Ref<br>◯ Missing<br>(frequency unit)
18. Margarine (either on food or for cooking or baking)<br><br>Enter number.<br><br>Enter unit in next question.<br>If Don't know, Refused, or Missing, enter in next question	______________________<br>(# times)
18. Margarine (either on food or for cooking or baking)<br><br>Enter unit	◯ Day<br>◯ Week<br>◯ Month<br>◯ Year<br>◯ Never<br>◯ Don't know<br>◯ Ref<br>◯ Missing<br>(frequency unit)
19. Vegetable oil (for example canola, corn, vegetable, safflower. olive) either on food or for cooking or baking<br><br>Enter number.<br><br>Enter unit in next question.<br>If Don't know, Refused, or Missing, enter in next question	______________________<br>(# times)

19. Vegetable oil (for example canola, corn, vegetable, safflower. olive) either on food or for cooking or baking

Enter unit

◯ Day
◯ Week
◯ Month
◯ Year
◯ Never
◯ Don't know
◯ Ref
◯ Missing
(frequency unit)

---

20. Potato chips

Enter number.

Enter unit in next question.
If Don't know, Refused, or Missing, enter in next question

____
(# times)

---

20. Potato chips

Enter unit

◯ Day
◯ Week
◯ Month
◯ Year
◯ Never
◯ Don't know
◯ Ref
◯ Missing
(frequency unit)

---

21. Other chips and snacks (tortilla chips, corn chips, cheese puffs, nuts, etc.)

Enter number.

Enter unit in next question.
If Don't know, Refused, or Missing, enter in next question

____
(# times)

---

21. Other chips and snacks (tortilla chips, corn chips, cheese puffs, nuts, etc.)

Enter unit

◯ Day
◯ Week
◯ Month
◯ Year
◯ Never
◯ Don't know
◯ Ref
◯ Missing
(frequency unit)

---

22. Microwave popcorn

Enter number.

Enter unit in next question.
If Don't know, Refused, or Missing, enter in next question

____
(# times)

22. Microwave popcorn<br><br>Enter unit	◯ Day<br>◯ Week<br>◯ Month<br>◯ Year<br>◯ Never<br>◯ Don't know<br>◯ Ref<br>◯ Missing<br>(frequency unit)
23. Movie theater popcorn<br><br>Enter number.<br><br>Enter unit in next question.<br>If Don't know, Refused, or Missing, enter in next question	____<br>(# times)
23. Movie theater popcorn<br><br>Enter unit	◯ Day<br>◯ Week<br>◯ Month<br>◯ Year<br>◯ Never<br>◯ Don't know<br>◯ Ref<br>◯ Missing<br>(frequency unit)
24. Sweets (cake, cookies, candy, chocolate)<br><br>Enter number.<br><br>Enter unit in next question.<br>If Don't know, Refused, or Missing, enter in next question	____<br>(# times)
24. Sweets (cake, cookies, candy, chocolate)<br><br>Enter unit	◯ Day<br>◯ Week<br>◯ Month<br>◯ Year<br>◯ Never<br>◯ Don't know<br>◯ Ref<br>◯ Missing<br>(frequency unit)
25. Fast Food and Take-Out Food<br><br>Enter number.<br><br>Enter unit in next question.<br>If Don't know, Refused, or Missing, enter in next question	____<br>(# times)

25. Fast Food and Take-Out Food

Enter unit

◯ Day
◯ Week
◯ Month
◯ Year
◯ Never
◯ Don't know
◯ Ref
◯ Missing
(frequency unit)

---

26. Take-out or delivered pizza

Enter number.

Enter unit in next question.
If Don't know, Refused, or Missing, enter in next question

____
(# times)

---

26. Take-out or delivered pizza

Enter unit

◯ Day
◯ Week
◯ Month
◯ Year
◯ Never
◯ Don't know
◯ Ref
◯ Missing
(frequency unit)

---

27. Food from a restaurant that is wrapped in paper or packaged in a cardboard container (for example hamburgers, sandwiches, burritos, Chinese food)

Enter number.

Enter unit in next question.
If Don't know, Refused, or Missing, enter in next question

____
(# times)

---

27. Food from a restaurant that is wrapped in paper or packaged in a cardboard container (for example hamburgers, sandwiches, burritos, Chinese food)

Enter unit

◯ Day
◯ Week
◯ Month
◯ Year
◯ Never
◯ Don't know
◯ Ref
◯ Missing
(frequency unit)

---

28. French fries (either from a take out or to-go restaurant)

Enter number.

Enter unit in next question.
If Don't know, Refused, or Missing, enter in next question

____
(# times)

28. French fries (either to-go or eaten at the restaurant)

Enter unit

◯ Day
◯ Week
◯ Month
◯ Year
◯ Never
◯ Don't know
◯ Ref
◯ Missing
(frequency unit)

29. Food from a store that comes in a paper or cardboard package and that you heat in the package, such as pizza, frozen meals, and garlic bread

Enter number.

Enter unit in next question.
If Don't know, Refused, or Missing, enter in next question

____
(# times)

29. Food from a store that comes in a paper or cardboard package and that you heat in the package, such as pizza, frozen meals, and garlic bread

Enter unit

◯ Day
◯ Week
◯ Month
◯ Year
◯ Never
◯ Don't know
◯ Ref
◯ Missing
(frequency unit)

The next questions I'm going to ask you are about non-stick or Teflon cookware. Non-stick coatings are smooth and usually black or gray.

31. How many non-stick or Teflon pots, pans or baking dishes do you use?

(For None, enter 0)
For Don't Know, enter 97.
For Ref, enter 98.
For Missing, enter 99.

____

32. How many of these non-stick pans and pots are scratched?

(For None, enter 0)
For Don't Know, enter 97.
For Ref, enter 98.
For Missing, enter 99.

____

33. How many times per day, week or month do you use your non-stick pans, pots or bake ware in the oven or broiler?

Enter number.

Enter unit in next question.
If Don't know, Refused, or Missing, enter in next question

____
(# times)

33. How many times per day, week or month do you use your non-stick pans, pots or bake ware in the oven or broiler?<br><br>Enter unit	◯ Day<br>◯ Week<br>◯ Month<br>◯ Year<br>◯ Never<br>◯ Don't know<br>◯ Ref<br>◯ Missing<br>(frequency unit)
34. Do you pre-heat your non-stick pans, pots or bake ware without food in them?	◯ No<br>◯ Yes<br>◯ Don't Know<br>◯ Ref<br>◯ Missing
35A. Do you have a rice cooker with a non-stick bowl?	◯ No<br>◯ Yes<br>◯ Don't Know<br>◯ Ref<br>◯ Missing
36A. How often do you use it?<br><br>Enter number.<br><br>Enter unit in next question.<br>If Don't know, Refused, or Missing, enter in next question	________________________<br>(# times)
36.A. How often do you use it?<br><br>Enter unit	◯ Day<br>◯ Week<br>◯ Month<br>◯ Year<br>◯ Never<br>◯ Don't know<br>◯ Ref<br>◯ Missing<br>(frequency unit)
37 A. How old is it?	◯ < 1 year<br>◯ 1-2 years<br>◯ 3-5 years<br>◯ >5 years
38.A. Does [ITEM] have any scratches?	◯ No<br>◯ Yes<br>◯ Don't know<br>◯ Ref<br>◯ Missing
39.A. Do you wash the [ITEM] in the dishwasher?	◯ No<br>◯ Yes<br>◯ Don't know<br>◯ Ref<br>◯ Missing

35.B. Do you have a non-stick electric grill or fry pan (like a George Foreman Grill)	◯ No<br>◯ Yes<br>◯ Don't know<br>◯ Ref<br>◯ Missing
36.B. How often do you use it?<br><br>Enter number.<br><br>Enter unit in next question.<br>If Don't know, Refused, or Missing, enter in next question	______________________<br>(# times)
36.B. How often do you use it?<br><br>Enter unit	◯ Day<br>◯ Week<br>◯ Month<br>◯ Year<br>◯ Never<br>◯ Don't know<br>◯ Ref<br>◯ Missing<br>(frequency unit)
37.B. How old is it?	◯ < 1 year<br>◯ 1-2 years<br>◯ 3-5 years<br>◯ >5 years
38.B. Does [ITEM] have any scratches?	◯ No<br>◯ Yes<br>◯ Don't know<br>◯ Ref<br>◯ Missing
35.C. Do you have a non-stick grilled sandwich maker or quesadilla	◯ No<br>◯ Yes - Sandwich<br>◯ Yes - Quesadilla<br>◯ Don't know<br>◯ Ref<br>◯ Missing
36.C. How often do you use it?<br><br>Enter number.<br><br>Enter unit in next question.<br>If Don't know, Refused, or Missing, enter in next question	______________________<br>(# times)
36.C. How often do you use it?<br><br>Enter unit	◯ Day<br>◯ Week<br>◯ Month<br>◯ Year<br>◯ Never<br>◯ Don't know<br>◯ Ref<br>◯ Missing<br>(frequency unit)

37.C. How old is it?	◯ < 1 year<br>◯ 1-2 years<br>◯ 3-5 years<br>◯ >5 years
38.C. Does [ITEM] have any scratches?	◯ No<br>◯ Yes<br>◯ Don't know<br>◯ Ref<br>◯ Missing
35.D. Do you have a non-stick waffle iron	◯ No<br>◯ Yes<br>◯ Don't know<br>◯ Ref<br>◯ Missing
36.D. How often do you use it?<br><br>Enter number.<br><br>Enter unit in next question.<br>If Don't know, Refused, or Missing, enter in next question	______________________<br>(# times)
36.D. How often do you use it?<br><br>Enter unit	◯ Day<br>◯ Week<br>◯ Month<br>◯ Year<br>◯ Never<br>◯ Don't know<br>◯ Ref<br>◯ Missing<br>(frequency unit)
37.D. How old is it?	◯ < 1 year<br>◯ 1-2 years<br>◯ 3-5 years<br>◯ >5 years
38.D. Does [ITEM] have any scratches?	◯ No<br>◯ Yes<br>◯ Don't know<br>◯ Ref<br>◯ Missing
35.E. Do you have a bread maker with a non-stick bowl	◯ No<br>◯ Yes<br>◯ Don't know<br>◯ Ref<br>◯ Missing
36.E. How often do you use it?<br><br>Enter number.<br><br>Enter unit in next question.<br>If Don't know, Refused, or Missing, enter in next question	______________________<br>(# times)

36.E. How often do you use it?<br><br>Enter unit	◯ Day<br>◯ Week<br>◯ Month<br>◯ Year<br>◯ Never<br>◯ Don't know<br>◯ Ref<br>◯ Missing<br>(frequency unit)
37.E. How old is it?	◯ < 1 year<br>◯ 1-2 years<br>◯ 3-5 years<br>◯ >5 years
38.E. Does [ITEM] have any scratches?	◯ No<br>◯ Yes<br>◯ Don't know<br>◯ Ref<br>◯ Missing
39.E. Do you wash the [ITEM] in the dishwasher?	◯ No<br>◯ Yes<br>◯ Don't know<br>◯ Ref<br>◯ Missing
44.A. During this pregnancy, have you treated any furniture, carpets or mattresses to be stain or water resistant?	◯ No<br>◯ Yes<br>◯ Don't Know<br>◯ Ref<br>◯ Missing
46.A. Did you or someone else do the work?	◯ Participant<br>◯ Someone else<br>◯ Both<br>◯ Don't Know<br>◯ Ref<br>◯ Missing
47.A. During your pregnancy, how many times have you been around when [FILL IN] including places other than your home?<br><br>If Never, enter 0.<br>If Don't Know, enter 97.<br>If Refused, enter 98.<br>If Missing, enter 99.	______________________________<br>(# times)
44.B. During this pregnancy, have you removed or installed any carpets	◯ No<br>◯ Yes - removed carpet<br>◯ Yes - installed carpet<br>◯ Don't Know<br>◯ Ref<br>◯ Missing

46.B. Did you or someone else do the work?

- ◯ Participant
- ◯ Someone else
- ◯ Both
- ◯ Don't Know
- ◯ Ref
- ◯ Missing

47.B. During your pregnancy, how many times have you been around when removing/installing carpets, including places other than your home?

____
(# times)

If Never, enter 0.
If Don't Know, enter 97.
If Refused, enter 98.
If Missing, enter 99.

44.C. During this pregnancy, have you had any furniture (tables, chairs, sofas, cabinets, book shelves, dressers) refinished or reupholstered

- ◯ No
- ◯ Yes - refinished furniture
- ◯ Yes - reupholstered furniture
- ◯ Don't Know
- ◯ Ref
- ◯ Missing

46.C. Did you or someone else do the work?

- ◯ Participant
- ◯ Someone else
- ◯ Both
- ◯ Don't Know
- ◯ Ref
- ◯ Missing

47.C. During your pregnancy, how many times have you been around when refinishing/reupholstering furniture, including places other than your home?

____
(# times)

If Never, enter 0.
If Don't Know, enter 97.
If Refused, enter 98.
If Missing, enter 99.

44.D. During this pregnancy, have you removed or installed any vinyl or linoleum flooring

- ◯ No
- ◯ Yes - removed flooring
- ◯ Yes - installed flooring
- ◯ Don't Know
- ◯ Ref
- ◯ Missing

46.D. Did you or someone else do the work?

- ◯ Participant
- ◯ Someone else
- ◯ Both
- ◯ Don't Know
- ◯ Ref
- ◯ Missing

47.D. During your pregnancy, how many times have you been around when removing/installing flooring, including places other than your home?

____
(# times)

If Never, enter 0.
If Don't Know, enter 97.
If Refused, enter 98.
If Missing, enter 99.

44.E. During this pregnancy, have you removed any wall paper?

- ◯ No
- ◯ Yes
- ◯ Don't Know
- ◯ Ref
- ◯ Missing

46.E. Did you or someone else do the work?

- ◯ Participant
- ◯ Someone else
- ◯ Both
- ◯ Don't Know
- ◯ Ref
- ◯ Missing

47.E. During your pregnancy, how many times have you been around when removing wall paper, including places other than your home?

____
(# times)

If Never, enter 0.
If Don't Know, enter 97.
If Refused, enter 98.
If Missing, enter 99.

44.F. During this pregnancy, have you caulked, grouted or sealed any windows or showers

- ◯ No
- ◯ Yes
- ◯ Don't Know
- ◯ Ref
- ◯ Missing

46.F. Did you or someone else do the work?

- ◯ Participant
- ◯ Someone else
- ◯ Both
- ◯ Don't Know
- ◯ Ref
- ◯ Missing

47.F. During your pregnancy, how many times have you been around when caulking, grouting or sealing any windows or showers, including places other than your home?

____
(# times)

If Never, enter 0.
If Don't Know, enter 97.
If Refused, enter 98.
If Missing, enter 99.

44.G. During this pregnancy, have you done any other home improvement work?	◯ No<br>◯ Yes<br>◯ Don't Know<br>◯ Ref<br>◯ Missing
45.G. Can you specify what type of home improvement work was done?	______________________
46.G. Did you or someone else do the work?	◯ Participant<br>◯ Someone else<br>◯ Both<br>◯ Don't Know<br>◯ Ref<br>◯ Missing
47.F. During your pregnancy, how many times have you been around when [homeother_specify], including places other than your home?<br><br>If Never, enter 0.<br>If Don't Know, enter 97.<br>If Refused, enter 98.<br>If Missing, enter 99.	______________________<br>(# times)
48. During this pregnancy, have you purchased or received any new upholstered furniture (furniture with fabric on it) or mattresses?	◯ No<br>◯ Yes<br>◯ Don't Know<br>◯ Ref<br>◯ Missing
49. How often do you open your window or use a fan when you shower or put on hair products, perfume or make up?	◯ Never<br>◯ Sometimes<br>◯ Often<br>◯ Always<br>◯ Don't Know<br>◯ Ref<br>◯ Missing
50. How often do you open a window or use a fan over your kitchen stove when you cook on your stove?	◯ Never<br>◯ Sometimes<br>◯ Often<br>◯ Always<br>◯ Don't Know<br>◯ Ref<br>◯ Missing
Questionnaire complete?	◯ No<br>◯ Yes<br>◯ Questionnaire missing
