# Supplementary material for: Relationships between psychosocial stressors among pregnant women in San Francisco: A path analysis
Source: PLoS One. 2020 Jun 12;15(6):e0234579. doi: 10.1371/journal.pone.0234579 (PMC7292353; doi:10.1371/journal.pone.0234579)
Supplement: S2 File — (DOCX) [file pone.0234579.s002.docx]

S2 File. Supporting tables for Relationships between psychosocial stressors among pregnant women in San Francisco: a path analysis

Table A. Summary of questions and scoring used to assess measures of psychosocial stress and response to stress.

| **Questionnaire** | **Questions and Potential Answers** | | **Scoring** |
| --- | --- | --- | --- |
| ***Dichotomous*** |  | |  |
| **Financial Strain** |  | |  |
|  | What is the combined family income before taxes? | | Household income below 2017 San Francisco county poverty line **OR** a. >3 |
|  | a. How hard is it for you to pay for very basics like food, housing, medical care, and heating?  *(1: Not difficult at all; 2: Not very difficult; 3: Somewhat difficult; 4: Very difficult.)* | |  |
| **Food Insecurity** |  | |  |
|  | *How often did this happen in your household in the last 12 months (1: Never True; 2: Sometimes True; 3: Often True).* | |  |
|  | | a. We couldn't afford to eat balanced and nutritious meals. | a>2 **OR** b>2 **OR** c+d+e>0 |
|  |  | b. The food that we bought just didn't last, and we didn't have money to get more. |  |
|  | *Did any of the following happen in your household in the last 12 months (0: No; 1: Yes).* | |  |
|  | | c. Adults ever cut or skip meals because there wasn't enough money for food? |  |
|  |  | d. Did you ever eat less than you felt you should because there wasn't enough money to buy food? |  |
|  |  | e. Were you ever hungry but didn't eat because you couldn't afford more food? |  |
| **Job Strain** |  | |  |
|  | *Thinking about your current job, how likely is each of the statements (1: Very unlikely; 2: Unlikely; 3: Neither likely nor unlikely; 4: Likely; 5: Very likely).* | |  |
|  | | a. My job allows me to make a lot of decisions on my own | (a<2 **OR** b>4 **OR** c<2) **AND** (d<2 **OR** e<2) |
|  | | b. I have an opportunity to develop my own special abilities. |  |
|  | | c. I am not asked to do an excessive amount of work. |  |
|  | | d. My job leaves me feeling too tired and stressed after work. |  |
|  | | e. Considering my efforts and achievements, my salary (pay) is fair. |  |
| **Discrimination** |  | |  |
|  | How often do you feel that you, personally, have been discriminated against because of your race, ethnicity, ancestry religion, or color? *(1: Never, 2: Rarely, 3: Sometimes, 4: Often, 5: Very often)* | | >4 |
| **Caregiving** |  | |  |
|  | *How often in the past 5 years (1: Never; 2: Rarely; 3: Sometimes; 4: Often; 5: Very often)* | |  |
|  | | a. Were you responsible for the care and well-being of a parent or any older relative? | a>4 **OR** b>4 |
|  | | b. Were you responsible for a child who needs more medical/health/educational services? |  |
| **Unplanned Pregnancy** |  | |  |
|  | Thinking back to just before you got pregnant, how did you feel about becoming pregnant? (*1: I didn't want to be pregnant then; 2: I wanted to be pregnant then; 3: I wanted to be pregnant later; 4: I wanted to be pregnant sooner.*) | | >3 |
| ***Continuous*** |  | |  |
| **Community Status** |  | |  |
|  | Where would you place yourself today, on a scale of 1 *(lowest)* - 10 *(highest)* standing in your community? | | 1-10 |
| **Depression** |  | |  |
|  | *In the past year, how often (0: Rarely; 1: Some of the time, 2: Often, 3: Most of the time)* | |  |
|  | | In the past year, how often were you bothered by things that usually do not bother you | Sum of all responses to each question |
|  | | In the past year, how often did you have trouble staying focused on what you were doing? |  |
|  | | In the past year, how often did you feel depressed? |  |
|  | | In the past year, how often did you feel that everything you did was an effort? |  |
|  | | *In the past year, how often did you feel hopeful about the future? |  |
|  | | In the past year, how often did you feel fearful? |  |
|  | | In the past year, how often was your sleep restless? |  |
|  | | *In the past year, how often were you happy? |  |
|  | | In the past year, how often did you feel lonely? |  |
|  | | *In the past year, how often did you feel that you could get going? |  |
| **Stressful Life Events** |  | |  |
|  | *Has any of the following happened during the last 12 months (1: Yes; 0: No).* | |  |
|  | | A close family member was very sick and had to go into the hospital | Sum of all responses to each question |
|  | | I got separated or divorced from my husband or partner |  |
|  | | I moved to a new address |  |
|  | | My spouse or partner lost his/her job |  |
|  | | I lost my job even though I wanted to go on working |  |
|  | | I argued with my spouse or partner more than usual |  |
|  | | My spouse or partner said s/he didn't want me to be pregnant |  |
|  | | I had a lot of bills I couldn't pay |  |
|  | | I was in a physical fight |  |
|  | | My spouse or partner had serious legal problems |  |
|  | | Someone very close to me had a problem with drinking or drugs |  |
|  | | Someone very close to me died |  |
|  | | I or a close family member has experienced immigration problems |  |
| **Perceived Stress** |  | |  |
|  | *How often in the past 5 years have you felt (0: Never; 1: Rarely; 2: Sometimes; 3: Often; 4: Very often):* | |  |
|  | | *That you were unable to control the important things in your life? | Sum of all responses to each question |
|  | | Confident about your ability to handle your personal problems? |  |
|  | | That things were going your way? |  |
|  | | *That difficulties were piling up so high that you could not overcome them? |  |
| **Neighborhood Quality** |  | |  |
|  | *Social Cohesion (1. Strongly disagree; 2: Somewhat disagree; 3: Neither agree nor disagree; 4: Somewhat agree; 5: Strongly agree)* | |  |
|  | | *People around here are willing to help their neighbors. | Sum of all responses to each question |
|  | | *This is a close-knit neighborhood. |  |
|  | | *People in this neighborhood can be trusted. |  |
|  | | People in this neighborhood generally don't get along with each other. |  |
|  | | People in this neighborhood don't share the same values. |  |
|  | *Informal Social Control (1. Strongly disagree; 2: Somewhat disagree; 3: Neither agree nor disagree; 4: Somewhat agree; 5: Strongly agree)* | |  |
|  | | Children were skipping school and hanging out on a street corner |  |
|  | | Children were spray-painting graffiti on a local building |  |
|  | | Children were showing disrespect to an adult |  |
|  | | A fight broke out in front of their house |  |
|  | *Neighborhood Safety (1. Strongly disagree; 2: Somewhat disagree; 3: Neither agree nor disagree; 4: Somewhat agree; 5: Strongly agree)* | |  |
|  | | *I feel safe in this neighborhood. |  |
|  | *Neighborhood Satisfaction (1. Strongly disagree; 2: Somewhat disagree; 3: Neither agree nor disagree; 4: Somewhat agree; 5: Strongly agree)* | |  |
|  | | *I think this neighborhood is a good place for me to live. |  |
|  | | I would move out of this neighborhood if I could. |  |
|  | *Physical Disorder (1. Strongly disagree; 2: Somewhat disagree; 3: Neither agree nor disagree; 4: Somewhat agree; 5: Strongly agree)* | |  |
|  | | There is a lot of loud noise from cars, motorcycles, music, neighbors, or airplanes in my neighborhood. |  |
|  | | My neighborhood has a lot of vacant lots or vacant houses. |  |
|  | | There is heavy car or truck traffic in this neighborhood. |  |

*Indicates positively worded statements that were reverse coded so that higher scores on each scale indicate higher stress.

Table B. Distribution of psychosocial stress measures across demographic characteristics and racial and ethnic groups.

|  | Caregiving | | Discrimination | | Financial Strain | | Job Strain | | Unplanned Pregnancy | | Food Insecurity | | Stressful Life Events | Neighborhood Quality |
| --- | --- | --- | --- | --- | --- | --- | --- | --- | --- | --- | --- | --- | --- | --- |
|  | Yes  N=78 (15%) | No  N=419 (82%) | Yes  N=35 (7%) | No  N=458 (90%) | Yes  N=174 (34%) | No  N=271 (53%) | Yes  N=65 (13%) | No  N=398 (78%) | Yes  N=139 (27%) | No  N=355 (70%) | Yes  N=81 (16%) | No  N=418 (82%) | Mean (SD) | Mean (SD) |
| **Maternal Education** |  |  |  |  |  |  |  |  |  |  |  |  |  |  |
| Less than High School | 15 (19.2) | 42 (10.0) | 19 (23.5) | 37 (8.9) | 41 (23.6) | 1 (0.4) | 18 (27.7) | 25 (6.3) | 24 (17.3) | 32 (9.0) | 19 (23.5) | 37 (8.9) | 2.58 (2.10) | 43.55 (10.71) |
| High School Degree or Some College | 38 (48.7) | 98 (23.4) | 52 (64.2) | 88 (21.1) | 93 (53.4) | 15 (5.5) | 26 (40.0) | 93 (23.4) | 59 (42.4) | 77 (21.7) | 52 (64.2) | 88 (21.1) | 2.84 (2.09) | 43.27 (11.39) |
| College Degree | 13 (16.7) | 105 (25.1) | 7 (8.6) | 110 (26.3) | 24 (13.8) | 89 (32.8) | 7 (10.8) | 108 (27.1) | 30 (21.6) | 87 (24.5) | 7 (8.6) | 110 (26.3) | 2.12 (1.71) | 38.86 (7.79) |
| Graduate Degree | 12 (15.4) | 172 (41.1) | 3 (3.7) | 181 (43.3) | 15 (8.6) | 165 (60.9) | 14 (21.5) | 170 (42.7) | 26 (18.7) | 157 (44.2) | 3 (3.7) | 181 (43.3) | 1.49 (1.25) | 37.74 (7.27) |
| **Maternal Race/Ethnicity** |  |  |  |  |  |  |  |  |  |  |  |  |  |  |
| Non-Hispanic White | 9 (11.5) | 184 (43.9) | 7 (8.6) | 187 (44.7) | 18 (10.3) | 172 (63.5) | 9 (13.8) | 182 (45.7) | 27 (19.4) | 164 (46.2) | 7 (8.6) | 187 (44.7) | 1.68 (1.54) | 37.94 (6.67) |
| Non-Hispanic Black | 12 (15.4) | 27 ( 6.4) | 13 (16.0) | 26 (6.2) | 23 (13.2) | 10 (3.7) | 8 (12.3) | 29 (7.3) | 18 (12.9) | 20 (5.6) | 13 (16.0) | 26 (6.2) | 3.08 (1.99) | 45.45 (12.50) |
| Hispanic | 42 (53.8) | 129 (30.8) | 52 (64.2) | 121 (28.9) | 107 (61.5) | 28 (10.3) | 43 (66.2) | 103 (25.9) | 72 (51.8) | 98 (27.6) | 52 (64.2) | 121 (28.9) | 2.63 (2.05) | 42.89 (10.23) |
| Asian/Pacific Islander | 15 (19.2) | 79 (18.9) | 9 (11.1) | 84 (20.1) | 26 (14.9) | 61 (22.5) | 5 (7.7) | 84 (21.1) | 22 (15.8) | 73 (20.6) | 9 (11.1) | 84 (20.1) | 1.82 (1.39) | 37.52 (8.87) |
| **Marital Status** |  |  |  |  |  |  |  |  |  |  |  |  |  |  |
| Married | 39 (50.0) | 295 (70.4) | 27 (33.3) | 310 (74.2) | 72 (41.4) | 243 (89.7) | 29 (44.6) | 292 (73.4) | 65 (46.8) | 267 (75.2) | 27 (33.3) | 310 (74.2) | 1.77 (1.58) | 38.46 (8.21) |
| Single | 37 (47.4) | 120 (28.6) | 54 (66.7) | 102 (24.4) | 99 (56.9) | 26 ( 9.6) | 35 (53.8) | 104 (26.1) | 72 (51.8) | 84 (23.7) | 54 (66.7) | 102 (24.4) | 2.96 (2.02) | 44.23 (10.72) |
| **Pre-pregnancy Body Mass Index** |  |  |  |  |  |  |  |  |  |  |  |  |  |  |
| Underweight (<18.5 kg/m^2^) | 1 (1.3) | 11 (2.6) | 2 (2.5) | 10 (2.4) | 1 (0.6) | 10 ( 3.7) | 1 (1.5) | 11 (2.8) | 1 (0.7) | 11 (3.1) | 2 (2.5) | 10 (2.4) | 1.83 (1.40) | 38.42 (5.11) |
| Normal Weight (18.5-24.9 kg/m^2^) | 24 (30.8) | 207 (49.4) | 22 (27.2) | 211 (50.5) | 45 (25.9) | 170 (62.7) | 21 (32.3) | 205 (51.5) | 58 (41.7) | 175 (49.3) | 22 (27.2) | 211 (50.5) | 1.82 (1.62) | 38.78 (9.02) |
| Overweight (25.0-29.9 kg/m^2^) | 31 (39.7) | 98 (23.4) | 24 (29.6) | 104 (24.9) | 59 (33.9) | 52 (19.2) | 21 (32.3) | 95 (23.9) | 41 (29.5) | 85 (23.9) | 24 (29.6) | 104 (24.9) | 2.56 (2.05) | 41.26 (8.69) |
| Obese (>30 kg/m^2^) | 18 (23.1) | 69 (16.5) | 27 (33.3) | 61 (14.6) | 55 (31.6) | 16 ( 5.9) | 18 (27.7) | 56 (14.1) | 33 (23.7) | 54 (15.2) | 27 (33.3) | 61 (14.6) | 2.33 (1.68) | 41.33 (10.42) |
| **Parity** |  |  |  |  |  |  |  |  |  |  |  |  |  |  |
| One or More Prior Births | 29 (37.2) | 214 (51.1) | 26 (32.1) | 216 (51.7) | 52 (29.9) | 174 (64.2) | 15 (23.1) | 222 (55.8) | 59 (42.4) | 181 (51.0) | 26 (32.1) | 216 (51.7) | 2.22 (1.77) | 39.92 (8.56) |
| No Prior Births | 47 (60.3) | 200 (47.7) | 21 (60.0) | 227 (49.6) | 120 (69.0) | 92 (33.9) | 49 (75.4) | 170 (42.7) | 79 (56.8) | 168 (47.3) | 54 (66.7) | 196 (46.9) | 2.09 (1.87) | 40.79 (10.12) |
| **Foreign Born** |  |  |  |  |  |  |  |  |  |  |  |  |  |  |
| Yes | 40 (51.3) | 165 (39.4) | 45 (55.6) | 162 (38.8) | 104 (59.8) | 67 (24.7) | 44 (67.7) | 135 (33.9) | 71 (51.1) | 135 (38.0) | 45 (55.6) | 162 (38.8) | 2.26 (1.88) | 41.17 (9.62) |
| No | 25 (32.1) | 185 (44.2) | 12 (34.3) | 197 (43.0) | 47 (27.0) | 151 (55.7) | 12 (18.5) | 195 (49.0) | 49 (35.3) | 159 (44.8) | 26 (32.1) | 186 (44.5) | 1.92 (1.66) | 39.59 (9.53) |

Abbreviations: SD, standard deviation.

Table C. Distribution of responses to psychosocial stress across demographic characteristics and racial and ethnic groups.

|  | **Perceived Stress** | **Depression** | **Community Status** |
| --- | --- | --- | --- |
|  | **Mean (SD)** | **Mean (SD)** | **Mean (SD)** |
| **Maternal Education** |  |  |  |
| Less than High School | 6.61 (2.98) | 10.25 (6.50) | 6.02 (2.09) |
| High School Degree or Some College | 6.62 (2.76) | 9.09 (6.12) | 5.70 (2.22) |
| College Degree | 4.62 (2.40) | 6.71 (4.32) | 6.52 (1.77) |
| Graduate Degree | 4.56 (2.14) | 5.51 (3.45) | 6.80 (1.48) |
| **Maternal Race/Ethnicity** |  |  |  |
| Non-Hispanic White | 4.62 (2.42) | 5.73 (3.96) | 6.82 (1.59) |
| Non-Hispanic Black | 6.90 (2.50) | 9.26 (5.25) | 6.36 (2.29) |
| Hispanic | 6.06 (2.82) | 8.69 (6.11) | 5.74 (2.14) |
| Asian/Pacific Islander | 5.01 (2.34) | 7.24 (4.56) | 6.48 (1.50) |
| **Marital Status** |  |  |  |
| Married | 4.80 (2.43) | 6.33 (4.41) | 6.48 (1.70) |
| Single | 6.55 (2.79) | 9.41 (6.09) | 6.14 (2.20) |
| **Pre-pregnancy Body Mass Index** |  |  |  |
| Underweight (<18.5 kg/m^2^) | 3.83 (1.90) | 4.92 (4.14) | 7.00 (0.89) |
| Normal Weight (18.5-24.9 kg/m^2^) | 5.00 (2.59) | 6.78 (4.80) | 6.59 (1.73) |
| Overweight (25.0-29.9 kg/m^2^) | 5.51 (2.76) | 8.00 (5.68) | 6.05 (2.19) |
| Obese (>30 kg/m^2^) | 5.99 (2.57) | 8.16 (5.49) | 6.28 (1.89) |
| **Parity** |  |  |  |
| One or More Prior Births | 5.22 (2.44) | 6.62 (4.47) | 6.53 (1.74) |
| No Prior Births | 5.49 (2.85) | 7.84 (6.54) | 6.18 (2.00) |
| **Foreign Born** |  |  |  |
| Yes | 5.70 (2.83) | 8.10 (5.81) | 5.89 (1.97) |
| No | 5.10 (2.41) | 6.62 (4.67) | 6.74 (1.67) |

Abbreviations: SD, standard deviation.

Table D. Regression coefficients and 95% confidence intervals for direct, indirect, and total effects between SES indicators and psychosocial stressor and stress response measures in the Chemicals in Our Bodies-2 cohort (N=258). Model has good fit (RMSEA=0.03, SRMR=0.052, CFI=1.00, TLI=0.99).

|  |  |  |  | **Direct Effect** | | **Indirect Effect** | | **Total Effect** | |
| --- | --- | --- | --- | --- | --- | --- | --- | --- | --- |
| **Independent Variable** | **Dependent Variable** | **Mediator Variable** | | **Beta** | **95% CI** | **Beta** | **95% CI** | **Beta** | **95% CI** |
| Stressful Life Events | Depression | Perceived Stress | | **0.73** | **(0.28, 1.18)** | **0.41** | **(0.22, 0.60)** | **1.14** | **(0.72, 1.56)** |
| Discrimination |  | Perceived Stress | | **7.34** | **(3.92, 10.75)** | 0.61 | (-1.00, 2.21) | **7.94** | **(3.97, 11.91)** |
| Neighborhood Quality |  | Perceived Stress | | 0.28 | (-0.39, 0.96) | -0.03 | (-0.33, 0.28) | 0.25 | (-0.47, 0.98) |
| Food Insecurity |  | Perceived Stress | | **6.02** | **(3.21, 8.83)** | 0.80 | (-0.34, 1.93) | **6.81** | **(3.69, 9.94)** |
| Job Strain |  | Perceived Stress | | **2.55** | **(0.27, 4.83)** | 0.82 | (-0.25, 1.89) | **3.37** | **(0.80, 5.94)** |
| Unplanned Pregnancy |  | Perceived Stress | | 1.41 | (-0.09, 2.91) | 0.66 | (-0.01, 1.34) | **2.08** | **(0.41, 3.74)** |
| Caregiving |  | – | | **4.37** | **(2.03, 6.71)** | – | – | **4.37** | **(2.03, 6.71)** |
| Financial Strain |  | – | | **3.70** | **(0.89, 6.52)** | **–** | **–** | **3.70** | **(0.89, 6.52)** |
| Perceived Stress |  | – | | **0.96** | **(0.66, 1.26)** | – | – | **0.96** | **(0.66, 1.26)** |
| Stressful Life Events | Perceived Stress | – | | **0.43** | **(0.24, 0.61)** | – | – | **0.43** | **(0.24, 0.61)** |
| Discrimination |  | – | | 0.63 | (-1.05, 2.30) | – | – | 0.63 | (-1.05, 2.30) |
| Neighborhood Quality |  | – | | -0.03 | (-0.35, 0.29) | – | – | -0.03 | (-0.35, 0.29) |
| Food Insecurity |  | – | | 0.83 | (-0.41, 2.07) | – | – | 0.83 | (-0.41, 2.07) |
| Job Strain |  | – | | 0.85 | (-0.25, 1.95) | – | – | 0.85 | (-0.25, 1.95) |
| Unplanned Pregnancy |  | – | | 0.69 | (-0.04, 1.42) | – | – | 0.69 | (-0.04, 1.42) |
| Stressful Life Events | Community Status | Perceived Stress | | – | – | -0.04 | (-0.08, 0.01) | -0.04 | (-0.08, 0.01) |
| Discrimination |  | Perceived Stress | | -1.03 | (-2.07, 0.02) | -0.06 | (-0.22, 0.10) | -1.09 | (-2.11, -0.06) |
| Neighborhood Quality |  | Perceived Stress | | **-0.53** | **(-0.78, -0.28)** | 0.00 | (-0.03, 0.03) | **-0.53** | **(-0.78, -0.28)** |
| Food Insecurity |  | Perceived Stress | | – | – | -0.08 | (-0.23, 0.08) | -0.08 | (-0.23, 0.08) |
| Job Strain |  | Perceived Stress | | – | – | -0.08 | (-0.22, 0.07) | -0.08 | (-0.22, 0.07) |
| Unplanned Pregnancy |  | Perceived Stress | | – | – | -0.06 | (-0.16, 0.04) | -0.06 | (-0.16, 0.04) |
| Perceived Stress |  | – | | -0.09 | (-0.19, 0.01) | – | – | -0.091 | (-0.19, 0.01) |

Note: Higher scores for Perceived Stress, Depression, and Neighborhood Quality indicate higher stressor and response levels. Lower scores for Community Status indicate higher stress response levels. Bold indicates statistical significance at p<0.05. Beta estimates for the direct effect correspond to S3 File Figure A and beta estimates for the direct, indirect, and total effect between SES indicators and psychosocial stress measures and responses to stress are provided in S2 File Table E-H.

Abbreviations: CI, confidence interval.

- Indicates no path

Table E. Regression coefficients and 95% confidence intervals for direct effects between SES indicators and psychosocial stressor and stress response measures in the Chemicals in Our Bodies-2 cohort (N=258). Model has good fit (RMSEA=0.03, SRMR=0.052, CFI=1.00, TLI=0.99).

|  |  | **Direct Effect** | |
| --- | --- | --- | --- |
| **Independent Variable**^a^ | **Dependent Variable** | **Beta** | **95% CI** |
| Education | Depression | **-9.55** | **(-14.38, -4.73)** |
| Race/Ethnicity |  | -0.06 | (-1.54, 1.42) |
| Foreign Born |  | -0.13 | (-1.59, 1.33) |
| Education | Perceived Stress | 0.82 | (-0.55, 2.20) |
| Race/Ethnicity |  | 0.17 | (-0.48, 0.82) |
| Foreign Born |  | -0.47 | (-1.14, 0.20) |
| Education | Community Status | -0.30 | (-1.02, 0.41) |
| Race/Ethnicity |  | 0.00 | (-0.49, 0.49) |
| Foreign Born |  | **-0.85** | **(-1.35, -0.36)** |
| Education | Stressful Life Events | **1.71** | **(1.07, 2.36)** |
| Race/Ethnicity |  | 0.10 | (-0.42, 0.61) |
| Foreign Born |  | -0.03 | (-0.53, 0.48) |
| Education | Discrimination | **0.16** | **(0.06, 0.26)** |
| Race/Ethnicity |  | -0.01 | (-0.08, 0.07) |
| Foreign Born |  | 0.00 | (-0.07, 0.08) |
| Education | Neighborhood Quality | **0.83** | **(0.55, 1.11)** |
| Race/Ethnicity |  | 0.02 | (-0.23, 0.26) |
| Foreign Born |  | -0.05 | (-0.33, 0.23) |
| Education | Food Insecurity | **0.48** | **(0.36, 0.61)** |
| Race/Ethnicity |  | **0.09** | **(0.01, 0.17)** |
| Foreign Born |  | -0.07 | (-0.17, 0.04) |
| Education | Job Strain | **0.21** | **(0.09, 0.32)** |
| Race/Ethnicity |  | 0.03 | (-0.05, 0.10) |
| Foreign Born |  | 0.08 | (-0.01, 0.18) |
| Education | Unplanned Pregnancy | **0.33** | **(0.19, 0.48)** |
| Race/Ethnicity |  | 0.05 | (-0.07, 0.17) |
| Foreign Born |  | 0.02 | (-0.11, 0.15) |
| Education | Caregiving | **0.30** | **(0.17, 0.43)** |
| Race/Ethnicity |  | 0.04 | (-0.04, 0.12) |
| Foreign Born |  | -0.01 | (-0.11, 0.09) |
| Education | Financial Strain | **0.79** | **(0.71, 0.88)** |
| Race/Ethnicity |  | **0.13** | **(0.02, 0.24)** |
| Foreign Born |  | 0.06 | (-0.05, 0.17) |

^a^SES indicators are coded as maternal race (non-Hispanic white yes/no), maternal education (<college degree, college or graduate degree), foreign born (yes/no born within the U.S.). Reference groups for SES indicators are as follows: Education- college or graduate degree. Race/ethnicity-non-Hispanic white. Foreign born-yes born within the U.S.

Note: Higher scores for Perceived Stress, Depression, and Neighborhood Quality indicate higher stressor and response levels. Lower scores for Community Status indicate higher stress response levels. Bold indicates statistical significance at p<0.05. Beta estimates for the direct effect correspond to S3 File Figure A and beta estimates for the indirect, and total effect between SES indicators and psychosocial stress measures and responses to stress are provided in S2 File Table E-H.

Abbreviations: CI, confidence interval.

Table F. Regression coefficients and 95% confidence intervals for indirect and total effects between education and psychosocial stressor and stress response measures in the Chemicals in Our Bodies-2 cohort (N=258). Model has good fit (RMSEA=0.03, SRMR=0.052, CFI=1.00, TLI=0.99).

|  |  |  | **Indirect Effect** | | **Total Effect** | |
| --- | --- | --- | --- | --- | --- | --- |
| **Independent Variable**^a^ | **Dependent Variable** | **Mediator Variable** | **Beta** | **95% CI** | **Beta** | **95% CI** |
| Education | Depression | Stressful Life Events | **1.25** | **(0.25, 2.24)** | **-8.31** | **(-12.69, -3.92)** |
|  |  | Discrimination | **1.18** | **(0.12, 2.23)** | **-8.38** | **(-12.80, -3.95)** |
|  |  | Neighborhood Quality | 0.23 | (-0.35, 0.82) | **-9.32** | **(-13.90, -4.75)** |
|  |  | Food Insecurity | **2.91** | **(1.2, 4.63)** | **-6.64** | **(-10.76, -2.52)** |
|  |  | Job Strain | 0.53 | (-0.09, 1.14) | **-9.03** | **(-13.67, -4.38)** |
|  |  | Unplanned Pregnancy | 0.47 | (-0.13, 1.07) | **-9.08** | **(-13.70, -4.46)** |
|  |  | Caregiving | **1.31** | **(0.25, 2.37)** | **-8.25** | **(-12.73, -3.76)** |
|  |  | Financial Strain | **2.94** | **(0.58, 5.3)** | **-6.61** | **(-10.59, -2.64)** |
|  |  | Perceived Stress | 0.79 | (-0.68, 2.26) | **-8.76** | **(-13.81, -3.72)** |
| Education | Depression | Stressful Life Events and Perceived Stress | **0.70** | **(0.27, 1.13)** | **-8.85** | **(-13.66, -4.04)** |
|  |  | Discrimination and Perceived Stress | 0.10 | (-0.17, 0.37) | -9.46 | (-14.26, -4.65) |
|  |  | Neighborhood Quality and Perceived Stress | -0.02 | (-0.28, 0.23) | **-9.58** | **(-14.42, -4.74)** |
|  |  | Food Insecurity and Perceived Stress | 0.38 | (-0.18, 0.95) | **-9.17** | **(-14.09, -4.25)** |
|  |  | Job Strain and Perceived Stress | 0.17 | (-0.06, 0.4) | **-9.39** | **(-14.2, -4.57)** |
|  |  | Unplanned Pregnancy and Perceived Stress | 0.22 | (-0.03, 0.47) | **-9.33** | **(-14.14, -4.53)** |
| Education | Community Status | Discrimination | -0.16 | (-0.38, 0.05) | -0.47 | (-1.14, 0.20) |
|  |  | Neighborhood Quality | **-0.44** | **(-0.7, -0.18)** | **-0.74** | **(-1.45, -0.04)** |
|  |  | Perceived Stress | -0.08 | (-0.21, 0.06) | -0.38 | (-1.08, 0.33) |
| Education | Community Status | Stressful Life Events and Perceived Stress | -0.07 | (-0.15, 0.02) | -0.07 | (-0.15, 0.02) |
|  |  | Discrimination and Perceived Stress | -0.01 | (-0.04, 0.02) | -0.31 | (-1.02, 0.4) |
|  |  | Neighborhood Quality and Perceived Stress | 0.00 | (-0.02, 0.03) | -0.30 | (-1.02, 0.41) |
|  |  | Food Insecurity and Perceived Stress | -0.04 | (-0.11, 0.04) | -0.34 | (-1.04, 0.36) |
|  |  | Job Strain and Perceived Stress | -0.02 | (-0.05, 0.02) | -0.32 | (-1.03, 0.39) |
|  |  | Unplanned Pregnancy and Perceived Stress | -0.02 | (-0.06, 0.01) | -0.32 | (-1.03, 0.38) |
| Education | Perceived Stress | Stressful Life Events | **0.73** | **(0.27, 1.19)** | **1.55** | **(0.27, 2.84)** |
|  |  | Discrimination | 0.10 | (-0.18, 0.38) | 0.92 | (-0.44, 2.28) |
|  |  | Neighborhood Quality | -0.03 | (-0.29, 0.23) | 1.00 | (-0.33, 2.32) |
|  |  | Food Insecurity | 0.40 | (-0.22, 1.02) | **1.22** | **(0.17, 2.28)** |
|  |  | Job Strain | 0.17 | (-0.07, 0.42) | 1.00 | (-0.33, 2.32) |
|  |  | Unplanned Pregnancy | 0.23 | (-0.04, 0.5) | 1.05 | (-0.24, 2.35) |

^a^SES indicators are coded as maternal race (non-Hispanic white yes/no), maternal education (<college degree, college or graduate degree), foreign born (yes/no born within the U.S.). Reference groups for SES indicators are as follows: Education- college or graduate degree. Race/ethnicity-non-Hispanic white. Foreign born-yes born within the U.S.

Note: Higher scores for Perceived Stress, Depression, and Neighborhood Quality indicate higher stressor and response levels. Lower scores for Community Status indicate higher stress response levels. Bold indicates statistical significance at p<0.05. Beta estimates for the direct effect between SES indicators and psychosocial stress measures and responses to stress are provided in S2 File Table E. Beta estimates for the indirect and total effect between SES indicators and psychosocial stress measures and responses to stress are provided in S2 File Table F-H.

Abbreviations: CI, confidence interval.

Table G. Regression coefficients and 95% confidence intervals for indirect and total effects between maternal race/ethnicity and psychosocial stressor and stress response measures in the Chemicals in Our Bodies-2 cohort (N=258). Model has good fit (RMSEA=0.03, SRMR=0.052, CFI=1.00, TLI=0.99).

|  | |  |  | **Indirect Effect** | | **Total Effect** | |
| --- | --- | --- | --- | --- | --- | --- | --- |
| **Independent Variable**^a^ | **Dependent Variable** | | **Mediator Variable** | **Beta** | **95% CI** | **Beta** | **95% CI** |
| Race/Ethnicity | Depression | | Stressful Life Events | 0.07 | (-0.31, 0.45) | 0.01 | (-1.33, 1.35) |
|  |  | | Discrimination | -0.04 | (-0.59, 0.51) | -0.10 | (-1.4, 1.21) |
|  |  | | Neighborhood Quality | 0.00 | (-0.07, 0.07) | -0.05 | (-1.51, 1.40) |
|  |  | | Food Insecurity | 0.53 | (-0.01, 1.08) | 0.47 | (-0.85, 1.80) |
|  |  | | Job Strain | 0.07 | (-0.13, 0.28) | 0.01 | (-1.46, 1.48) |
|  |  | | Unplanned Pregnancy | 0.07 | (-0.11, 0.26) | 0.01 | (-1.43, 1.45) |
|  |  | | Caregiving | 0.17 | (-0.18, 0.53) | 0.11 | (-1.25, 1.48) |
|  |  | | Financial Strain | 0.48 | (-0.08, 1.05) | 0.42 | (-0.81, 1.66) |
|  |  | | Perceived Stress | 0.16 | (-0.47, 0.79) | 0.10 | (-1.53, 1.74) |
| Race/Ethnicity | Depression | | Stressful Life Events and Perceived Stress | 0.04 | (-0.17, 0.25) | -0.02 | (-1.41, 1.37) |
|  |  | | Discrimination and Perceived Stress | 0.00 | (-0.05, 0.04) | -0.06 | (-1.52, 1.39) |
|  |  | | Neighborhood Quality and Perceived Stress | 0.00 | (-0.01, 0.01) | -0.06 | (-1.54, 1.42) |
|  |  | | Food Insecurity and Perceived Stress | 0.07 | (-0.04, 0.18) | 0.01 | (-1.44, 1.46) |
|  |  | | Job Strain and Perceived Stress | 0.02 | (-0.05, 0.09) | -0.04 | (-1.51, 1.43) |
|  |  | | Unplanned Pregnancy and Perceived Stress | 0.03 | (-0.05, 0.12) | -0.02 | (-1.48, 1.44) |
| Race/Ethnicity | Community Status | | Discrimination | 0.01 | (-0.07, 0.08) | 0.01 | (-0.48, 0.49) |
|  |  | | Neighborhood Quality | -0.01 | (-0.14, 0.12) | -0.01 | (-0.49, 0.48) |
|  |  | | Perceived Stress | -0.02 | (-0.08, 0.05) | -0.01 | (-0.50, 0.48) |
| Race/Ethnicity | Community Status | | Stressful Life Events and Perceived Stress | 0.00 | (-0.02, 0.02) | 0.00 | (-0.49, 0.49) |
|  |  | | Discrimination and Perceived Stress | 0.00 | (0.00, 0.00) | 0.00 | (-0.49, 0.49) |
|  |  | | Neighborhood Quality and Perceived Stress | 0.00 | (0.00, 0.00) | 0.00 | (-0.49, 0.49) |
|  |  | | Food Insecurity and Perceived Stress | -0.01 | (-0.02, 0.01) | 0.00 | (-0.49, 0.48) |
|  |  | | Job Strain and Perceived Stress | 0.00 | (-0.01, 0.00) | 0.00 | (-0.49, 0.49) |
|  |  | | Unplanned Pregnancy and Perceived Stress | 0.00 | (-0.01, 0.01) | 0.00 | (-0.49, 0.49) |
| Race/Ethnicity | Perceived Stress | | Stressful Life Events | 0.04 | (-0.17, 0.26) | 0.21 | (-0.47, 0.89) |
|  |  | | Discrimination | 0.00 | (-0.05, 0.05) | 0.17 | (-0.49, 0.82) |
|  |  | | Neighborhood Quality | 0.00 | (-0.01, 0.01) | 0.17 | (-0.48, 0.82) |
|  |  | | Food Insecurity | 0.07 | (-0.05, 0.20) | 0.24 | (-0.4, 0.89) |
|  |  | | Job Strain | 0.02 | (-0.05, 0.10) | 0.19 | (-0.46, 0.85) |
|  |  | | Unplanned Pregnancy | 0.04 | (-0.05, 0.12) | 0.21 | (-0.45, 0.86) |

^a^SES indicators are coded as maternal race (non-Hispanic white yes/no), maternal education (<college degree, college or graduate degree), foreign born (yes/no born within the U.S.). Reference groups for SES indicators are as follows: Education- college or graduate degree. Race/ethnicity-non-Hispanic white. Foreign born-yes born within the U.S.

Note: Higher scores for Perceived Stress, Depression, and Neighborhood Quality indicate higher stressor and response levels. Lower scores for Community Status indicate higher stress response levels. Beta estimates for the direct effect between SES indicators and psychosocial stress measures and responses to stress are provided in S2 File Table E. Beta estimates for the indirect and total effect between SES indicators and psychosocial stress measures and responses to stress are provided in S2 File Table F-SH.

Abbreviations: CI, confidence interval.

Table H. Regression coefficients and 95% confidence intervals for direct, indirect, and total effects between foreign born and psychosocial stressor and stress response measures in the Chemicals in Our Bodies-2 cohort (N=258). Model has good fit (RMSEA=0.03, SRMR=0.052, CFI=1.00, TLI=0.99).

|  |  |  | **Indirect Effect** | | **Total Effect** | |
| --- | --- | --- | --- | --- | --- | --- |
| **Independent Variable**^a^ | **Dependent Variable** | **Mediator Variable** | **Beta** | **95% CI** | **Beta** | **95% CI** |
| Foreign Born | Depression | Stressful Life Events | -0.02 | (-0.39, 0.34) | -0.15 | (-1.50, 1.20) |
|  |  | Discrimination | 0.01 | (-0.53, 0.56) | -0.12 | (-1.43, 1.20) |
|  |  | Neighborhood Quality | -0.01 | (-0.11, 0.08) | -0.14 | (-1.58, 1.30) |
|  |  | Food Insecurity | -0.40 | (-1.04, 0.25) | -0.53 | (-1.89, 0.83) |
|  |  | Job Strain | 0.21 | (-0.09, 0.51) | 0.08 | (-1.35, 1.51) |
|  |  | Unplanned Pregnancy | 0.03 | (-0.15, 0.22) | -0.10 | (-1.52, 1.32) |
|  |  | Caregiving | -0.04 | (-0.47, 0.40) | -0.17 | (-1.53, 1.19) |
|  |  | Financial Strain | 0.22 | (-0.19, 0.63) | 0.09 | (-1.23, 1.41) |
|  |  | Perceived Stress | -0.45 | (-1.11, 0.20) | -0.58 | (-2.25, 1.08) |
| Foreign Born | Depression | Stressful Life Events and Perceived Stress | -0.01 | (-0.22, 0.20) | -0.14 | (-1.53, 1.25) |
|  |  | Discrimination and Perceived Stress | 0.00 | (-0.04, 0.05) | -0.13 | (-1.57, 1.31) |
|  |  | Neighborhood Quality and Perceived Stress | 0.00 | (-0.01, 0.02) | -0.13 | (-1.59, 1.34) |
|  |  | Food Insecurity and Perceived Stress | -0.05 | (-0.17, 0.06) | -0.18 | (-1.62, 1.25) |
|  |  | Job Strain and Perceived Stress | 0.07 | (-0.05, 0.18) | -0.06 | (-1.52, 1.39) |
|  |  | Unplanned Pregnancy and Perceived Stress | 0.02 | (-0.07, 0.10) | -0.11 | (-1.55, 1.32) |
| Foreign Born | Community Status | Discrimination | 0.00 | (-0.08, 0.07) | **-0.81** | **(-1.30, -0.32)** |
|  |  | Neighborhood Quality | 0.03 | (-0.12, 0.17) | **-0.83** | **(-1.35, -0.31)** |
|  |  | Perceived Stress | 0.04 | (-0.04, 0.12) | **-0.81** | **(-1.30, -0.32)** |
| Foreign Born | Community Status | Stressful Life Events and Perceived Stress | 0.00 | (-0.02, 0.02) | **-0.85** | **(-1.35, -0.36)** |
|  |  | Discrimination and Perceived Stress | 0.00 | (0.00, 0.00) | **-0.85** | **(-1.35, -0.36)** |
|  |  | Neighborhood Quality and Perceived Stress | 0.00 | (0.00, 0.00) | **-0.85** | **(-1.35, -0.36)** |
|  |  | Food Insecurity and Perceived Stress | 0.00 | (-0.01, 0.02) | **-0.85** | **(-1.34, -0.36)** |
|  |  | Job Strain and Perceived Stress | -0.01 | (-0.02, 0.01) | **-0.86** | **(-1.35, -0.37)** |
|  |  | Unplanned Pregnancy and Perceived Stress | 0.00 | (-0.01, 0.01) | **-0.86** | **(-1.35, -0.36)** |
| Foreign Born | Perceived Stress | Stressful Life Events | 0.00 | (-0.05, 0.05) | -0.47 | (-1.13, 0.19) |
|  |  | Discrimination | 0.00 | (-0.05, 0.05) | -0.47 | (-1.13, 0.19) |
|  |  | Neighborhood Quality | 0.00 | (-0.02, 0.02) | -0.47 | (-1.14, 0.20) |
|  |  | Food Insecurity | 0.00 | (-0.05, 0.05) | -0.47 | (-1.13, 0.19) |
|  |  | Job Strain | 0.07 | (-0.05, 0.19) | -0.40 | (-1.06, 0.26) |
|  |  | Unplanned Pregnancy | 0.02 | (-0.08, 0.11) | -0.45 | (-1.12, 0.21) |

^a^SES indicators are coded as maternal race (non-Hispanic white yes/no), maternal education (<college degree, college or graduate degree), foreign born (yes/no born within the U.S.). Reference groups for SES indicators are as follows: Education- college or graduate degree. Race/ethnicity-non-Hispanic white. Foreign born-yes born within the U.S.

Note: Higher scores for Perceived Stress, Depression, and Neighborhood Quality indicate higher stressor and response levels. Lower scores for Community Status indicate higher stress response levels. Beta estimates for the direct effect between SES indicators and psychosocial stress measures and responses to stress are provided in S2 File Table E. Beta estimates for the indirect and total effect between SES indicators and psychosocial stress measures and responses to stress are provided in S2 File Table F-H.

Abbreviations: CI, confidence interval.
