# Supplementary figures and images for: Relationships between psychosocial stressors among pregnant women in San Francisco: A path analysis
Source: PLoS One. 2020 Jun 12;15(6):e0234579. doi: 10.1371/journal.pone.0234579 (PMC7292353; doi:10.1371/journal.pone.0234579)

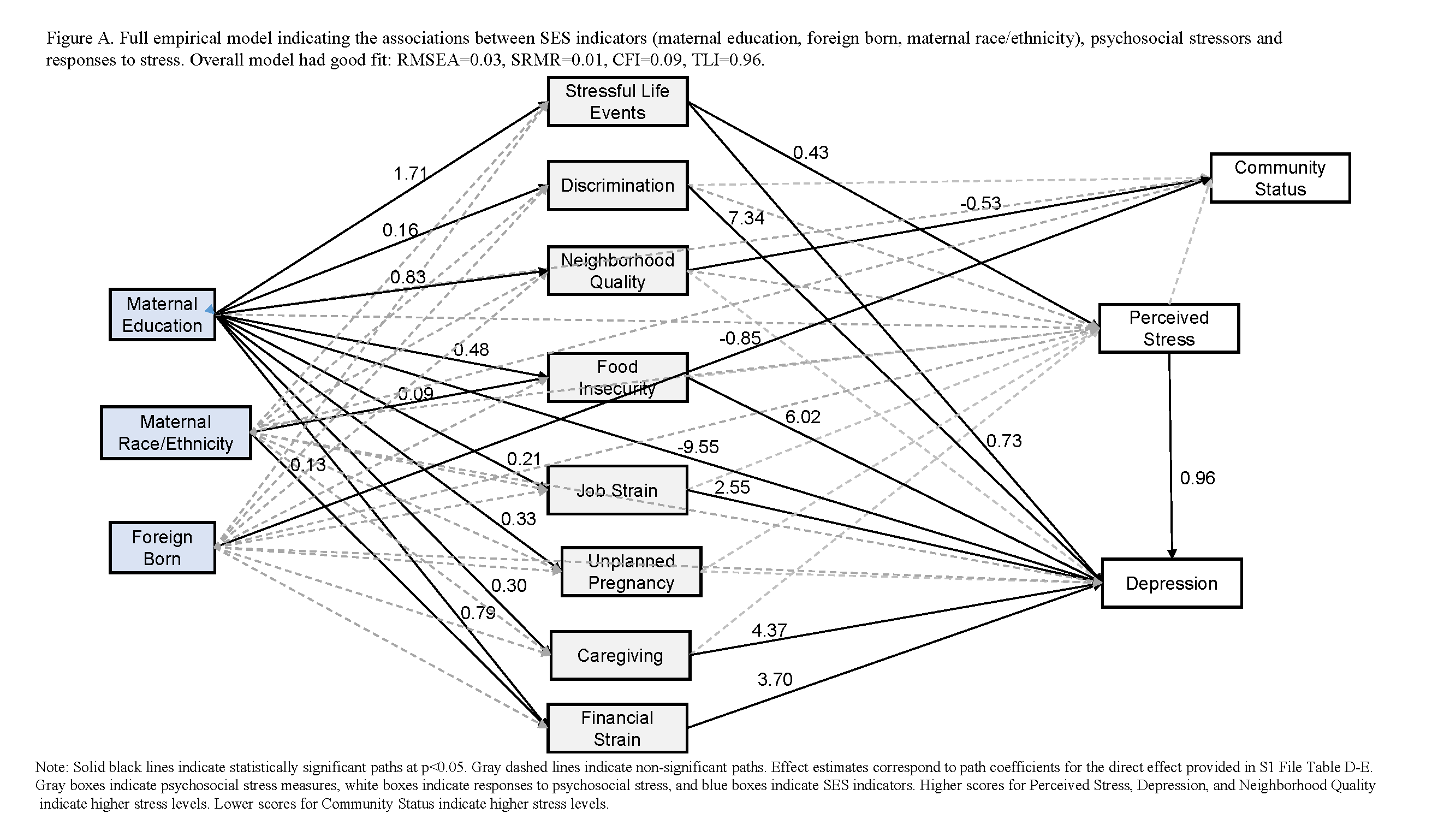

Supplement: S3 File — (TIFF) [file pone.0234579.s003.tiff]
